# Sirtuin 6 is a key contributor to gender differences in acute kidney injury

**DOI:** 10.1038/s41420-023-01432-y

**Published:** 2023-04-25

**Authors:** Jinhua Miao, Jiewu Huang, Ye Liang, Yunfang Zhang, Jiemei Li, Ping Meng, Weiwei Shen, Xiaolong Li, Qinyu Wu, Xiaoxu Wang, Hongxin Niu, Ying Tang, Shan Zhou, Lili Zhou

**Affiliations:** 1 State Key Laboratory of Organ Failure Research, National Clinical Research Center of Kidney Disease, Guangdong Provincial Clinical Research Center for Kidney Disease, Guangdong Provincial Key Laboratory of Nephrology, Division of Nephrology, Nanfang Hospital, Southern Medical University, Guangzhou, China; 2 Department of Nephrology, Huadu District People’s Hospital, Southern Medical University, Guangzhou, China; 3 Department of General Practice, Special Medical Service Center, Zhujiang Hospital, Southern Medical University, Guangzhou, China; 4 Department of Nephrology, The Third Affiliated Hospital of Southern Medical University, Guangzhou, China

**Keywords:** Apoptosis, Nuclear receptors

## Abstract

Acute kidney injury (AKI) is rapidly increasing nowadays and at a high risk to progress into chronic kidney disease (CKD). Of note, men are more susceptive to AKI, suggesting gender differences in AKI patients. However, the underlying mechanisms remain largely unclear. To test it, we adopted two experimental models of AKI, including ischemia/reperfusion injury and rhabdomyolysis, which were constructed in age-matched male and female mice. We found severe damages of tubular apoptosis, mitochondrial dysfunction, and loss of renal function showing in male mice, while female mice only had very mild injury. We further tested the expression of Sirtuins, and found that female mice could preserve more Sirtuin members’ expression in case of kidney damage. Among Sirtuin family, Sirtuin 6 was maximally preserved in injured kidney in female mice, suggesting its important role involved in the gender differences of AKI pathogenesis. We then found that knockdown of androgen receptor (AR) attenuated tubular damage, mitochondrial dysfunction and retarded the loss of renal function. Overexpression of Sirtuin 6 also showed similar results. Furthermore, in cultured tubular cells, dihydrotestosterone (DHT) decreased Sirtuin 6 expression and exacerbated cell apoptosis. Ectopic expression of Sirtuin 6 sufficiently inhibited DHT-induced cell apoptosis. Mechanically, we found AR inhibited Sirtuin 6, leading to the repression of binding of Sirtuin 6 with PGC-1α. This resulted in acetylation of PGC-1α and inhibition of its activity, further triggered the loss of mitochondrial homeostasis. Our results provided new insights to the underlying mechanisms of gender differences in AKI, suggesting Sirtuin 6 maybe a new therapeutic target for preventing AKI in male patients.

## Introduction

AKI could be induced by multifactorial factors such as ischemia or toxicity [1], and is characterized by a rapid decline of renal function and high incidence to progress into CKD. Nowadays, the incidence of AKI is rapidly increasing worldwide [2, 3]. Epidemical investigation showed, regardless of the multiple pathogenesis of AKI involving sepsis, ischemia-reperfusion injury (IRI), or various nephrotoxins [1], men are more susceptible to suffer from AKI [4, 5]. Indeed, the gender differences occur in many diseases such as pulmonary hypertension [6], autoimmune diseases [7] and neurodegenerative diseases [8, 9], as well as AKI [10]. Several reports have shown that sex hormones may play important roles in gender differences in the prevalence of diseases [4, 5, 11]. However, the underlying mechanisms of gender differences in the pathogenesis of AKI have not been clarified in detail.

Renal tubular epithelial cells (TECs) are the main constituent of the renal parenchyma. For actively undertaking reabsorption, secretion and excretion, TECs have huge energy requirement, hence, they are most vulnerable after injury [12, 13]. Mitochondria are the most important organelles for adenosine triphosphate (ATP) production [14]. Due to the huge energy requirement of TECs, mitochondrial homeostasis are highly associated with TECs stability and normal renal function [14]. Reports have shown dysfunctional mitochondria are heavily accumulated in renal TECs in AKI [15], suggesting the important role of mitochondrial homeostasis in therapeutic strategies for AKI. Of note, mitochondrial dysfunction contributes to ATP deficiency, impaired fatty acid metabolism and excessive reactive oxygen species (ROS) production, which cooperatively lead to the activation of caspase-3 signaling pathway and tubular cell apoptosis, a major phenomenon in AKI injury [1, 16–20].

Apoptosis, a type of the programmed cell death, in renal TECs, is the most common pathological feature in AKI [21, 22]. Notably, apoptotic cells exhibit the activation of catabolic proteases enzymes in cells, resulting in the destruction of cellular structures and induction of diseases [23]. Actually, large quantities of reports have demonstrated that TEC apoptosis plays a vital role in the pathogenesis of AKI [1, 21, 24]. However, the underlying mechanisms of tubular cell apoptosis still need to be elucidated in detail.

Androgen and estrogen are the main components of sex hormones. Recent reports showed that renal cell apoptosis may highly relate with sex hormone, especially androgen. Androgen mainly includes testosterone and dihydrotestosterone (DHT). Testosterone is the major circulating form of androgen and can be metabolized into DHT, a more potent form of androgen. Compared to testosterone, DHT has ten-fold higher affinity to AR [25]. AR could also serve as a transcriptional factor [26] to deeply involve into cell proliferation, migration, invasion, differentiation and apoptosis [26, 27]. Reports showed that testosterone is intimately related with renal tubular cell apoptosis, suggesting the potent role of androgen in the development of AKI [28]. However, the underlying mechanisms of AR in AKI are still in mystery.

Sirtuin family, belonging to class III histone deacetylases, is comprised of seven members, Sirtuin 1~7 [29]. Sirtuins are highly involved in maintaining cellular proliferation, DNA repair, mitochondrial energy homeostasis, antioxidant activity, and anti-apoptosis function [30, 31]. Sirtuins are localized in different subcellular compartments [31]. Sirtuin 1, 6, and 7 are located in nucleus respectively, Sirtuin 3, 4, and 5 are inside of mitochondria [32], and Sirtuin 2 is in cytosolic compartment [31]. Studies have reported that Sirtuin 1 plays a protective role in preserving mitochondrial homeostasis in AKI via deacetylating and activating proliferator‐activated receptor‐γ coactivator‐1α (PGC‐1α), a master regulator of mitochondrial biogenesis [31, 33, 34]. However, it is unknown whether Sirtuin 6, also localizing in nucleus like Sirtuin 1, can also de-acetylate and activate PGC-1α, and further preserve mitochondrial function in AKI. The other report also showed Sirtuin 3 could be involved in sex differences in AKI [35]. Furthermore, reports have shown that several members of Sirtuin family such as Sirtuin 1 and Sirtuin 3, could be modulated by androgen-mediated signaling pathway [36]. However, the authentic role of Sirtuin in gender differences in AKI still should be clarified in detail.

In this study, it showed more severe mitochondrial dysfunction, tubular cell apoptosis and kidney injury in male mice, compared with female mice under AKI condition. Among Sirtuin members, Sirtuin 6 was decreased maximally in male mice and largely preserved in female mice. Mechanically, AR induces Sirtuin 6 repression, leading to PGC-1α acetylation, which further triggered mitochondrial dysfunction. Our results provided a new therapeutic target for resolving the mess of gender-sensitive AKI prevalence.

## Results

### Male mice are more susceptive to tubular injury in IRI model

**Fig. 1:**
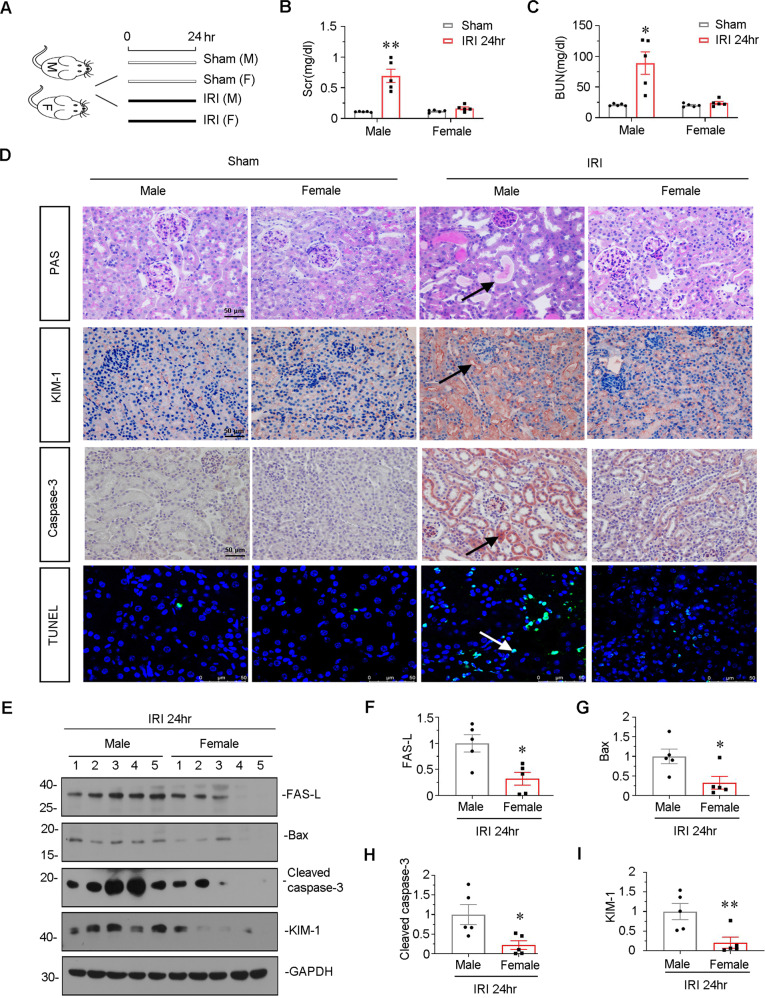
Male mice were more susceptive to IRI and tubular apoptosis in kidney. **A** Experimental design. Female and male mice were subjected to IRI or sham respectively, and euthanized 24 h after IRI. **B** Scr levels in four groups, as indicated. Scr was expressed as milligrams per deciliter. **P < 0.01 versus sham controls in male group (*n* = 5). **C** BUN levels in four groups, as indicated. BUN was expressed as milligrams per deciliter. *P < 0.05 versus sham controls in male group (*n* = 5). **D** Representative micrographs show renal tubular morphologic injury, the expression of KIM-1 and caspase-3, and TUNEL assay in different groups, as indicated. Paraffin sections were subjected to periodic acid–Schiff (PAS) staining, stained with an antibody against KIM-1 and caspase-3. Frozen kidney sections were subjected to TUNEL assay. Arrows indicate positive staining. Scale bar, 50 μm. **E**–**I** Representative western blot (**E**) and graphical representations of (**F**) FAS-L, (**G**) Bax, (**H**) cleaved caspase-3 and (**I**) KIM-1 protein expression levels are shown. *P < 0.05, **P < 0.01 versus male group (*n* = 5).

To investigate the gender difference, we constructed IRI model in both male and female mice. The experimental design is shown in Fig. 1A. As shown in Fig. 1B, C, Scr and BUN levels were significantly upregulated after renal IRI in male mice, but not in female mice. We then performed PAS staining to test tubular injury of dilation, injury, and casts [37]. As shown (Fig. 1D), IRI surgery induced a typical morphologic change in male mice. However, female mice showed very weak damage. We then performed the immunostaining of kidney injury molecule 1 (KIM-1), a biomarker for renal proximal tubule injury [38]. As shown in Fig. 1D, compared with female mice, male mice exhibited higher expression of KIM-1 in tubular epithelial cells (TECs) upon IRI. As tubular cell apoptosis is the main phenomenon in AKI, we then tested it in both IRI-induced male and female mice. We performed immunohistochemistry of caspase-3, an effector molecule in the process of apoptosis [39] and TUNEL assay, a diagnostic golden standard measure of cellular apoptosis [40]. As shown, in IRI-induced male mice, caspase-3 was strongly induced in tubules, however, there is only extremely slight expression of caspase-3 in female mice (Fig. 1D). Consistently, the number of TUNEL positive tubular cells significantly increased in IRI-induced male mice, in comparison with female mice (Fig. 1D). Furthermore, the western blotting analysis showed the consistent results. We found that compared with male mice, IRI could only induce a very weak expression of apoptosis-related proteins such as FAS-L, Bax, and the active form of caspase-3 [39, 41–43], and KIM-1(Fig. 1E–I). These results suggested male mice were more susceptible to apoptosis and IRI, which maybe explain the underlying mechanisms of gender differences in AKI.

### Sirtuin 6 is a contributor to gender difference upon IRI

**Fig. 2:**
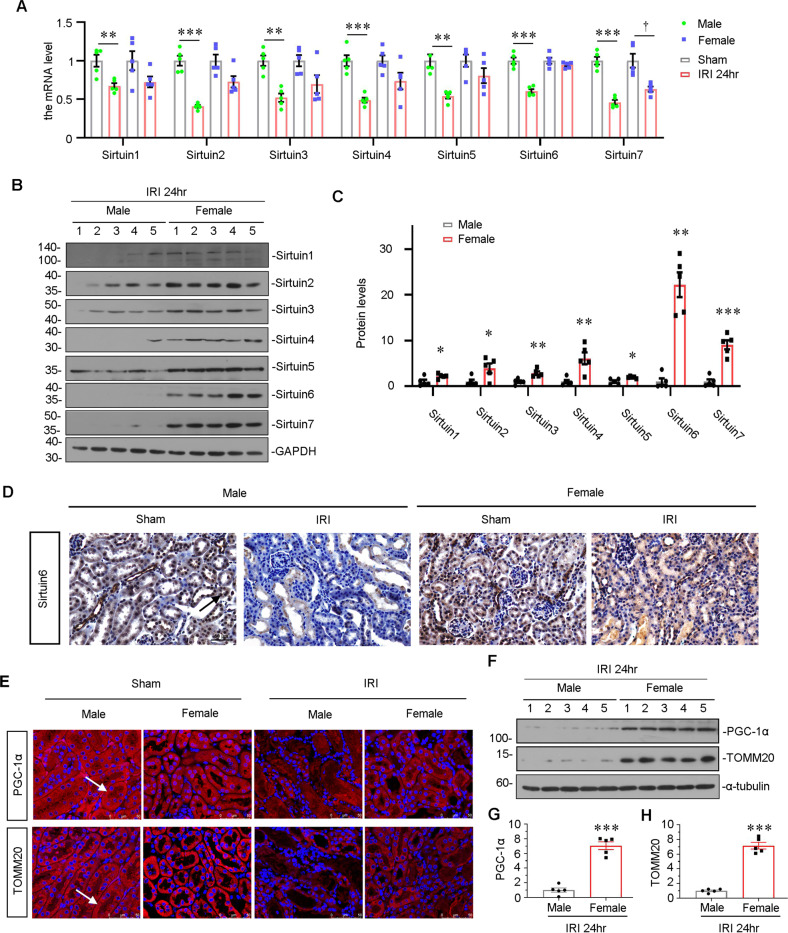
Sirtuin 6 is the possible contributor to gender differences upon IRI. **A** Graphical representations show the relative abundance of Sirtuin 1-7 mRNA in different groups. **P < 0.01, ***P < 0.001 versus sham controls in male group (*n* = 5); ^†^*P* < 0.05 versus sham controls in female group (*n* = 5). **B**, **C** Representative western blot (**B**) and graphical representations of (**C**) Sirtuin 1-7 protein expression levels are shown. *P < 0.05, **P < 0.01, ***P < 0.001 versus male group (*n* = 5). **D** Representative micrographs showing the expression of Sirtuin 6 in different groups, as indicated. Paraffin-embedded kidney sections were stained with an antibody against Sirtuin 6. Arrows indicate positive staining. Scale bar, 50 μm. **E** Representative micrographs showing the expression of PGC-1α and TOMM20 in different groups, as indicated. Frozen kidney sections were stained with an antibody against PGC-1α and TOMM20. Arrows indicate positive staining. Scale bar, 50 μm. **F**–**H** Representative western blot (**F**) and graphical representations of (**G**) PGC-1α and (**H**) TOMM20 protein expression levels are shown. ***P < 0.001 versus male group (*n* = 5).

Sirtuin family is highly related with sex hormone signaling. Hence, we further investigated the expression of Sirtuin family members. The mRNA levels of all Sirtuin members were assessed in both male and female mice. As shown in Fig. 2A, all Sirtuin members significantly decreased in male IRI mice, compared with sham control. But there were only slight decreases in female IRI mice compared with their sham control. Notably, in female mice, among all Sirtuin members, Sirtuin 6 was nearly completely preserved after IRI injury, suggesting its potential role in contributing to gender differences in AKI. We then performed western blotting. As shown in Supplementary Fig. 1A, B, the basal protein expression of all Sirtuin members was similar in female and male mice. However, as shown in Fig. 2B, C, compared with male IRI mice, all Sirtuin members were preserved to some extents in female IRI mice, especially Sirtuin 6, which was extraordinarily preserved. Consistently, the immunostaining showed Sirtuin 6 was greatly preserved in female IRI mice, compared to the extreme low expression of Sirtuin 6 in male IRI mice (Fig. 2D). These results suggested that Sirtuin 6 was possibly a contributor to gender differences upon IRI.

We then tested the mitochondrial function, which plays an important role in tubular cell apoptosis and the progression of AKI [15, 44]. In male IRI mice, the expression of translocase of outer mitochondrial membrane complex subunit 20 (TOMM20) [45], an outer mitochondrial membrane protein, and mitochondrial biogenesis‐related transcription factor PGC‐1α, a master regulator regulating mitochondrial biogenesis [33], were greatly lost in renal TECs (Fig. 2E). However, their expression was largely preserved in female IRI mice. The western blot analysis also showed TOMM20 and PGC‐1α were reserved in female mice after IRI surgery (Fig. 2F–H).

### Male mice exhibited more severe injury in rhabdomyolysis-induced AKI

**Fig. 3:**
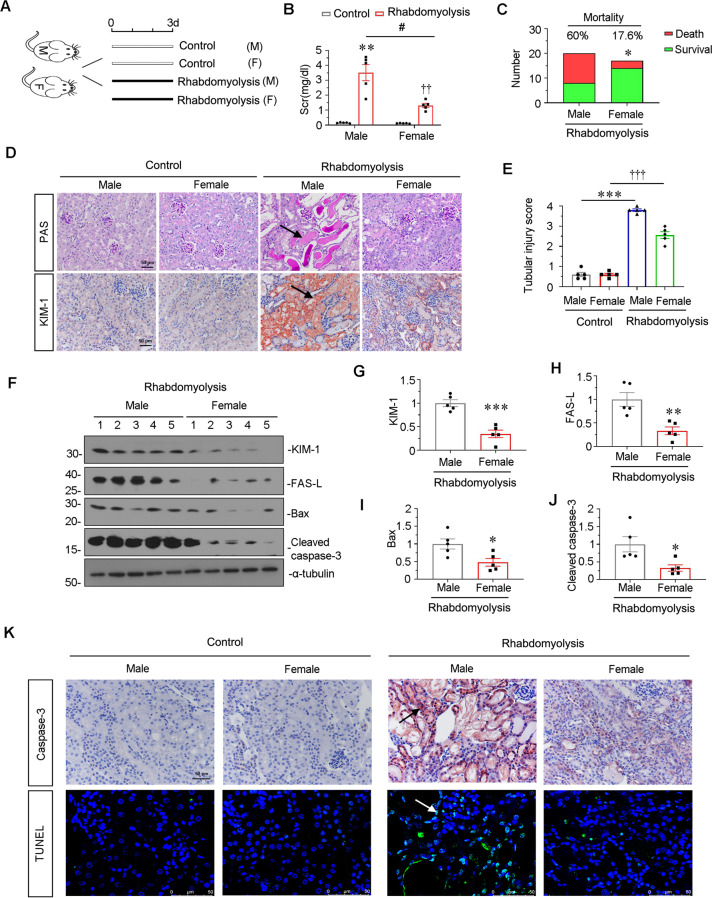
Male mice were more susceptive to rhabdomyolysis-induced AKI and tubular apoptosis in kidney. **A** Experimental design. Female and male mice were intramuscularly injected with 50% glycerol at the dose of 7.5 ml/kg or normal saline respectively. Mice were euthanized 3 days after intramuscular injection. **B** Scr levels in four groups, as indicated. Scr was expressed as milligrams per deciliter. **P < 0.01 versus sham controls in male group (*n* = 5); ^††^*P* < 0.01 versus sham controls in female group (*n* = 5); ^#^*P* < 0.05 versus male group in glycerol group (*n* = 5). **C** Graphical representations show three day-mortality in different genders after glycerol administration, as indicated. *P < 0.05 versus male group (n of male group = 20; *n* of female group = 17). **D** Representative micrographs show renal tubular morphologic injury and the expression of KIM-1 in different groups, as indicated. Paraffin sections were subjected to PAS staining and stained with an antibody against KIM-1. Arrows indicate positive staining. Scale bar, 50 μm. **E** Tubular injury score depending on PAS staining in four groups, as indicated. ***P < 0.001 versus sham controls in male group (*n* = 5); ^†††^*P* < 0.001 versus sham controls in female group (*n* = 5). **F**–**J** Representative western blot (**F**) and graphical representations of (**G**) KIM-1, (**H**) FAS-L, (**I**) Bax and (**J**) cleaved caspase-3 protein expression levels are shown. *P < 0.05, **P < 0.01, ***P < 0.001 versus female group (*n* = 5). **K** Representative micrographs show the expression of caspase-3 and TUNEL staining in different groups, as indicated. Paraffin sections were stained with an antibody against caspase-3. Frozen kidney sections were subjected to TUNEL staining. Arrow indicates positive staining. Scale bar, 50 μm.

We further constructed rhabdomyolysis-induced AKI mice model in both male and female mice. The experimental design is shown in Fig. 3A. As shown in Fig. 3B, the levels of Scr were significantly increased in male mice, but showed only slight increase in female mice. Moreover, the mortality of male mice after rhabdomyolysis-induced AKI reached 60%, but it was only 17.6% in female mice (Fig. 3C). We then performed PAS staining and immunostaining of KIM-1. As shown in Fig. 3D, E, in male mice, rhabdomyolysis induced very severe tubular injury, characterized with tubular dilation, cell loss and cast formation, while there was only mild injury in female mice. Similar results were also observed when KIM-1 was observed by immunostaining. We next performed western blotting. Male mice exhibited higher expression of KIM-1 and apoptosis-related proteins such as FAS-L, Bax, cleaved caspase-3, in comparison with female (Fig. 3F–J). Consistently, as shown in Fig. 3K, immunostaining and TUNEL staining also showed higher expression of caspase-3 and more TUNEL + cells in male mice, but there was only very mild change in female mice.

### Sirtuin 6 is the key regulator for gender differences in rhabdomyolysis-induced AKI

**Fig. 4:**
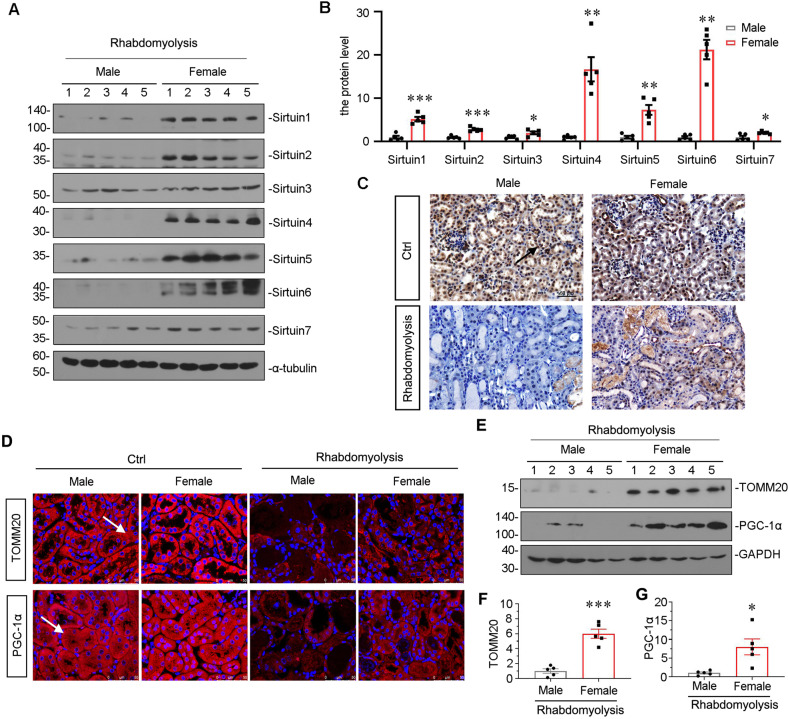
The expression of Sirtuin 6 was the key regulator for gender differences in rhabdomyolysis-induced AKI. **A**, **B** Representative western blot (**A**) and graphical representations of (**B**) Sirtuin 1-7 protein expression levels are shown. *P < 0.05, **P < 0.01, ***P < 0.001 versus female group (*n* = 5). **C** Representative micrographs show the expression of Sirtuin 6 in different groups, as indicated. Paraffin-embedded kidney sections were stained with an antibody against Sirtuin 6. Arrows indicate positive staining. Scale bar, 50 μm. **D** Representative micrographs showing the expression of PGC-1α and TOMM20 in different groups, as indicated. Frozen kidney sections were stained with an antibody against PGC-1α and TOMM20. Arrows indicate positive staining. Scale bar, 50 μm. **E**–**G** Representative western blot (**E**) and graphical representations of (**F**) PGC-1α and (**G**) TOMM20 protein expression levels are shown. *P < 0.05, ***P < 0.001 versus male group (*n* = 5).

We next assessed the expression of Sirtuin family. As shown in Fig. 4A, B, the whole family of Sirtuin was well preserved in rhabdomyolysis-induced female mice, especially Sirtuin 6. We then performed immunostaining. As shown in Fig. 4C, compared to sham control mice, there was a huge loss of Sirtuin 6 in male mice after rhabdomyolysis injury, however, Sirtuin 6 was perfectly preserved in female mice. Furthermore, we assessed the expression of TOMM20 and PGC-1α via immunofluorescence staining. As shown in Fig. 4D, compared with the great loss in male mice, TOMM20 and PGC-1α were largely preserved in female mice in rhabdomyolysis-induced AKI model. The similar results were observed when TOMM20 and PGC-1α were assessed by western blot analysis (Fig. 4E–G).

### Knockdown of AR ameliorates kidney injury and preserves mitochondrial homeostasis upon IRI injury

**Fig. 5:**
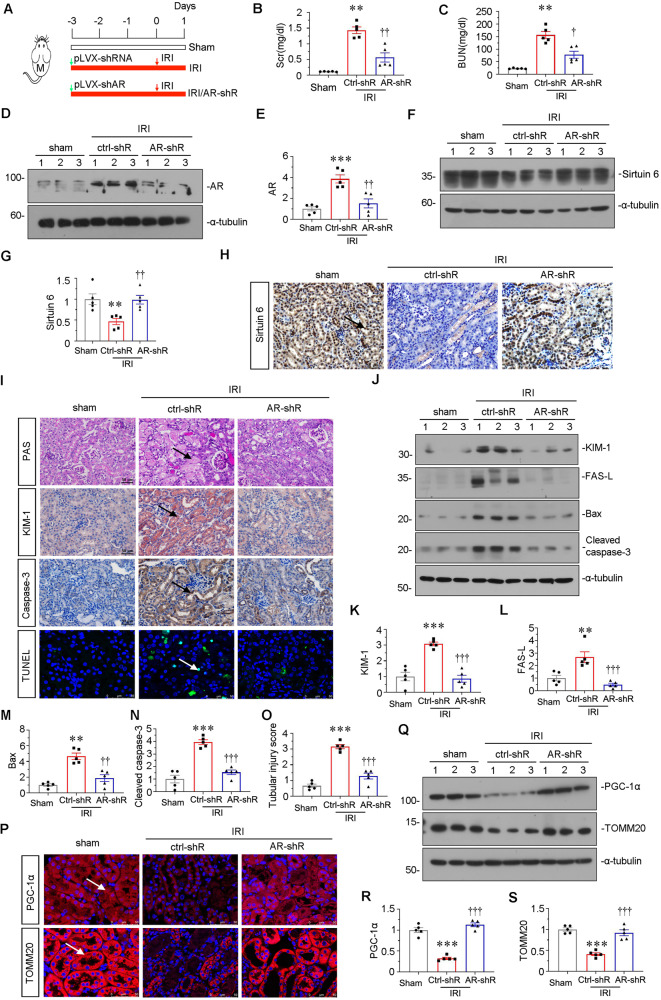
The ectopic knockdown of AR ameliorates renal injury and mitochondrial dysfunction upon IRI. **A** Experimental design. Green arrow showed the injection of control-shRNA (pLVX-shRNA) or AR-shRNA (pLVX-shAR) plasmid. Male mice were subjected to IRI or sham respectively, and euthanized 24 h after IRI. **B** Scr levels in three groups, as indicated. Scr was expressed as milligrams per deciliter. **P < 0.01 versus sham controls (*n* = 5); ^††^*P* < 0.01 versus control-shRNA group (*n* = 5). **C** BUN levels in three groups, as indicated. BUN was expressed as milligrams per deciliter. **P < 0.01 versus sham controls (*n* = 5); ^†^*P* < 0.05 versus control-shRNA group (*n* = 5). **D**, **E** Representative western blot (**D**) and graphical representations of (**E**) AR protein expression levels are shown. ***P < 0.001 versus sham controls (*n* = 5); ^††^*P* < 0.01 versus control-shRNA group (*n* = 5). **F**, **G** Representative western blot (**F**) and graphical representations of (**G**) Sirtuin 6 protein expression levels are shown. **P < 0.01 versus sham controls (*n* = 5); ^††^*P* < 0.01 versus control-shRNA group (*n* = 5). **H** Representative micrographs showing the expression of Sirtuin 6 in different groups. Paraffin sections were stained with an antibody against Sirtuin 6. Arrows indicate positive staining. Scale bar, 50 μm. **I** Representative micrographs show renal tubular morphologic injury, the expression of KIM-1 and caspase-3, and TUNEL assay in different groups, as indicated. Paraffin sections were subjected to PAS staining, stained with an antibody against KIM-1 and caspase-3. Frozen kidney sections were subjected to TUNEL staining. Arrows indicate positive staining. Scale bar, 50 μm. **(J**–**N)** Representative western blot (**J**) and graphical representations of (**K**) KIM-1, (**L**) FAS-L, (**M**) Bax and (**N**) cleaved caspase-3 protein expression levels are shown. **P < 0.01, ***P < 0.001 versus sham controls (*n* = 5); ^††^*P* < 0.01, ^†††^*P* < 0.001 versus control-shRNA group (*n* = 5). **O** Tubular injury scores depending on PAS staining in three groups, as indicated. ***P < 0.001 versus sham controls (*n* = 5); ^†††^*P* < 0.001 versus control-shRNA group (*n* = 5). **P** Representative micrographs showing the expression of PGC-1α and TOMM20 in different groups, as indicated. Frozen kidney sections were stained with an antibody against PGC-1α and TOMM20. Arrows indicate positive staining. Scale bar, 50 μm. **Q**–**S** Representative western blot (**Q**) and graphical representations of (**R**) PGC-1α and (**S**) TOMM20 protein expression levels are shown. ***P < 0.001 versus sham controls (*n* = 5); ^†††^*P* < 0.001 versus control-shRNA group (*n* = 5).

Due to the exhibition of more severe kidney injury after AKI in male mice, we hypothesized the androgen plays a key role in AKI development. Hence, we injected a shRNA-mediated AR plasmid to knockdown AR in IRI mice. The experimental design was shown in Fig. 5A. As shown in Fig. 5B, C, knockdown of AR significantly alleviated the increase in Scr and BUN levels in IRI mice. We then performed western bolt to verify the effects of AR knockdown. As shown in Fig. 5D, E, the expression of AR was upregulated in IRI male mice, while AR-shRNA injection effectively downregulated AR protein expression in IRI male mice. Moreover, we found that knockdown of AR could greatly restore the expression of Sirtuin 6 in renal tubules in IRI mice (Fig. 5F–H). Then we performed PAS staining and immunohistochemistry of KIM-1 to assess kidney injury. As shown in Fig. 5I, IRI surgery induced severe damages in tubular cells, while knockdown of AR strongly inhibited these effects. We then assessed tubular apoptosis through immunostaining for caspase-3 and TUNEL assay. As shown, knockdown of AR blocked the expression of caspase-3 and decreased TUNEL + cells (Fig. 5I). Furthermore, western blot analysis showed knockdown of AR significantly inhibited the expression of KIM-1, FAS-L, Bax and cleaved caspase-3 in IRI mice (Fig. 5J–N). The quantification of tubular cell injury also showed the deficiency of AR greatly inhibited tubular injury (Fig. 5O). We then assessed mitochondrial homeostasis. As shown in Fig. 5P–S, knockdown of AR strongly preserved the expression of TOMM20 and PGC-1α in TECs upon IRI.

### The ectopic expression of Sirtuin 6 relieves renal injury and mitochondrial dysfunction upon IRI

**Fig. 6:**
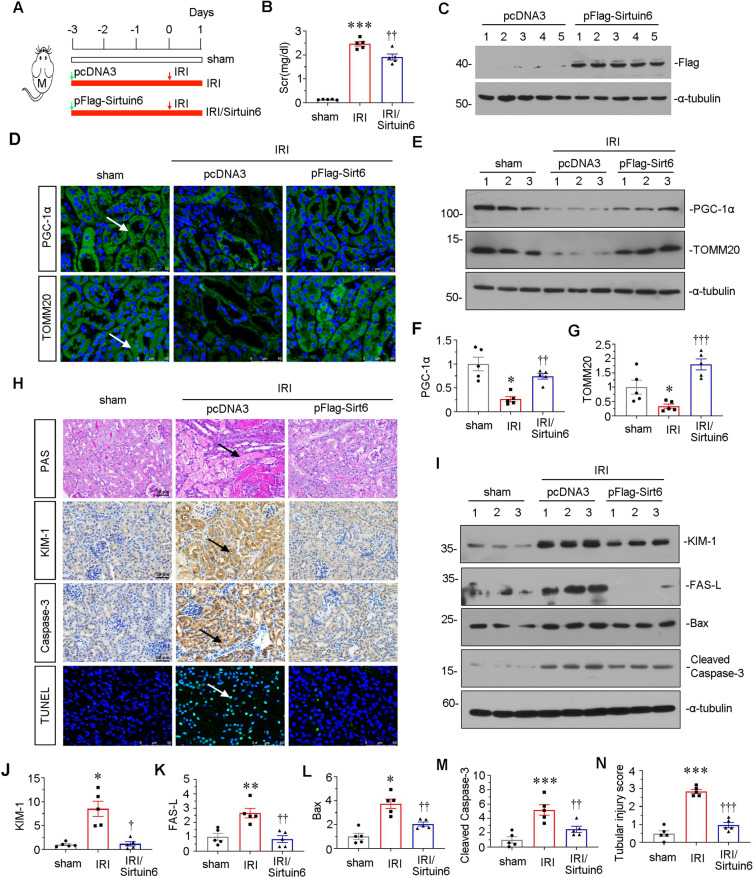
The ectopic expression of Sirtuin 6 relieves renal injury and mitochondrial dysfunction upon IRI. **A** Experimental design. Green arrow showed the injection of pcDNA plasmid or pFlag-Sirtuin 6 overexpression plasmid. Male mice were subjected to IRI or sham respectively, and euthanized 24 h after IRI. **B** Scr levels in three groups, as indicated. Scr was expressed as milligrams per deciliter. ***P < 0.001 versus sham controls (*n* = 5); ^††^*P* < 0.01 versus pcDNA group (*n* = 5). **C** Representative western blot of flag tag is shown. **D** Representative micrographs showing the expression of PGC-1α and TOMM20 in different groups, as indicated. Frozen kidney sections were stained with an antibody against PGC-1α and TOMM20. Arrows indicate positive staining. Scale bar, 50 μm. **E**–**G** Representative western blot (**E**) and graphical representations of (**F**) PGC-1α and (**G**) TOMM20 protein expression levels are shown. *P < 0.05 versus sham controls (*n* = 5); ^††^*P* < 0.01, ^†††^*P* < 0.001 versus pcDNA group (*n* = 5). **H** Representative micrographs show renal tubular morphologic injury, the expression of KIM-1 and caspase-3, and TUNEL assay in different groups, as indicated. Paraffin sections were subjected to periodic acid–Schiff (PAS) staining, stained with an antibody against KIM-1 and caspase-3. Frozen kidney sections were subjected to TUNEL staining. Arrows indicate positive staining. Scale bar, 50 μm. **I**–**M** Representative western blot (**I**) and graphical representations of (**J**) KIM-1, (**K**) FAS-L, (**L**) Bax and (**M**) cleaved caspase-3 protein expression levels are shown. *P < 0.05, **P < 0.01, ***P < 0.001 versus sham controls (*n* = 5); ^†^*P* < 0.05, ^††^*P* < 0.01 versus pcDNA group (*n* = 5). **N** Tubular injury score depending on PAS staining in three groups, as indicated. ***P < 0.001 versus sham controls (*n* = 5); ^†††^*P* < 0.001 versus pcDNA group (*n* = 5).

To further investigate the role of Sirtuin 6 in IRI injury, we injected Sirtuin 6 expressing plasmid into IRI mice. The experimental design is shown in Fig. 6A. As shown in Fig. 6B, ectopic expression of Sirtuin 6 significantly decreased the level of Scr in IRI mice. The successful expression of Sirtuin 6 was also demonstrated (Fig. 6C). We then assessed the expression of PGC-1α and TOMM20. As shown in Fig. 6D–G, their expression was inhibited in IRI mice, but largely preserved in Sirtuin 6-overexpressed IRI mice. We next tested tubular injury and cell apoptosis. PAS staining showed IRI-induced tubular dilation, cast formation, and cell loss were greatly inhibited by ectopic expression of Sirtuin 6 (Fig. 6H). Immunostaining of KIM-1 also demonstrated Sirtuin 6 could greatly ameliorate tubular cell injury (Fig. 6H). We then analyzed cell apoptosis. As shown, the increased expression of caspase-3 and TUNEL + cells in IRI mice were strongly blocked in Sirtuin 6-overexpressed group. Similar results were observed when KIM-1, FAS-L, Bax, and cleaved caspase-3 were observed by western blot analysis (Fig. 6I–M). In addition, quantification of tubular cell injury also revealed that Sirtuin 6 could greatly protect against AKI (Fig. 6N).

### AR transcriptionally downregulates Sirtuin 6 and induces acetylation of PGC-1α

**Fig. 7:**
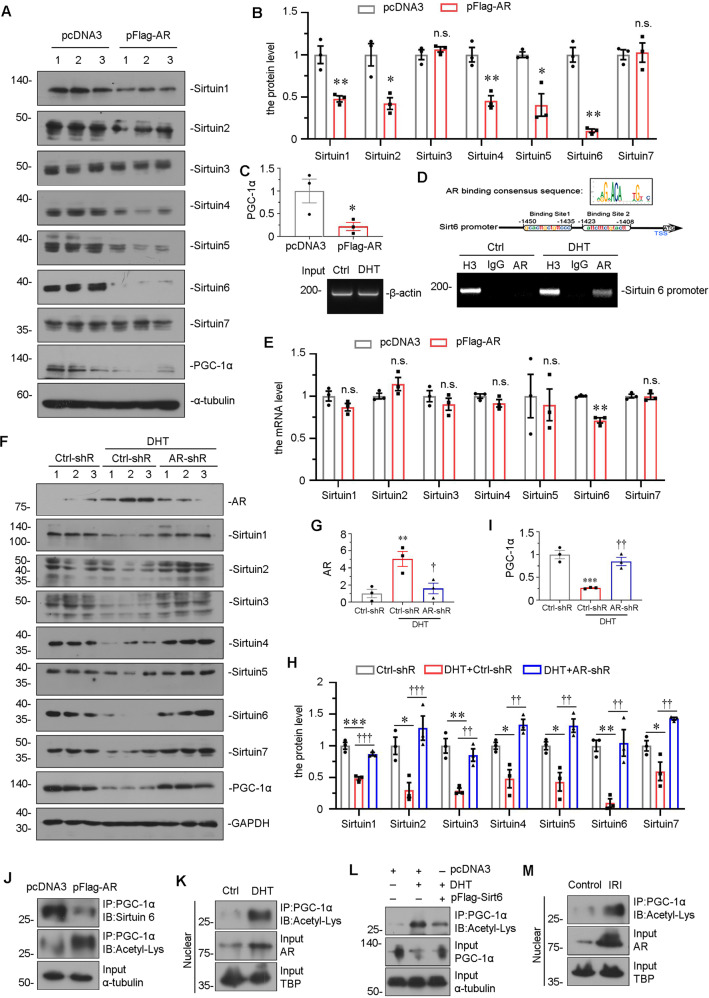
AR increases acetylation of PGC-1α by downregulating Sirtuin 6 expression. **A**–**C** Representative western blot (**A**) and graphical representations of (**B**) Sirtuin 1-7 and (**C**) PGC-1α protein expression levels are shown. *P < 0.05, **P < 0.01 versus control group (*n* = 3). HKC-8 cells were transfected with pcDNA3 or AR overexpression plasmid for 24 h. **D** Representative ChIP assay results showing the binding of AR to the Sirtuin 6 gene promoter region. HKC‐8 cells were incubated with DHT (10 μMol/L) or not for 24 h. Cell lysates were precipitated with an antibody against AR, histone H3, or nonimmune IgG, and the ChIP assay was performed for Sirtuin 6 gene promoters. Total diluted lysate was used as the total genomic input DNA. **E** Graphical representations show the relative abundance of Sirtuin 1-7 mRNA in different groups. **P < 0.01 versus control group (*n* = 3). **F**–**I** Representative western blot (**F**) and graphical representations of (**G**) Sirtuin 1-7, (**H**) PGC-1α and (**I**) AR protein expression levels are shown. HKC-8 cells were incubated with DHT (10 μMol/L) and transfected with AR-shRNA for 24 h. *P < 0.05, **P < 0.01, ***P < 0.001 (*n* = 3); ^†^*P* < 0.05, ^††^*P* < 0.01, ^†††^*P* < 0.001 (*n* = 3). **J** Representative graphs show the binding of PGC-1α with Sirtuin 6 or acetyl. HKC-8 cells were transfected with pcDNA3 or AR overexpression plasmid for 24 h. **K** Representative graphs show the binding of PGC-1α with acetyl, and the expression of PGC-1α in different groups, as indicated. HKC-8 cells were treated with DHT (10 μMol/L) and transfected with Sirtuin 6 overexpression plasmid for 24 h. **L** Representative graphs show the binding of PGC-1α with acetyl, and the protein levels of AR in nuclear fractions in different groups, as indicated. HKC-8 cells were treated with DHT (10 μMol/L) for 24 h. **M** Representative graphs show the binding of PGC-1α with acetyl, and the protein levels of AR in nuclear fractions in sham control and IRI group in male mice, as indicated.

We then cultured HKC-8 cells, a human proximal tubular cell line. HKC-8 cells were transfected with AR-overexpression plasmid, and whole-cell lysis was assessed the expression of Sirtuin family and PGC-1α. As shown in Fig. 7A, B, AR overexpression almost reduced the expression of all members of Sirtuins, especially Sirtuin 6, as well as inhibited the expression of PGC-1α (Fig. 7C). From bioinformatics analysis, we found that there is a binding site of AR on Sirtuin 6 promoter sequence. To testify their binding, we performed ChIP analysis in DHT-stimulated HKC-8 cells. As shown in Fig. 7D, we found a perfect biding of AR on Sirtuin 6 promoter region. We then performed PCR analysis. As shown, overexpression of AR decreased the mRNA level of Sirtuin 6, but not other Sirtuin members (Fig. 7E). These results suggest the direct regulation of AR on Sirtuin 6.

We then transfected HKC-8 with AR shRNA and then treated cells with DHT. As shown, DHT significantly increased the expression of AR, and reduced the expression of PGC-1α and Sirtuins, especially Sirtuin 6, while AR knockdown largely inhibited these effects (Fig. 7F–I). To investigate the correlations among AR, Sirtuin 6 and PGC-1α, we performed immunoprecipitation assay. HKC-8 cells were transfected with AR expressing plasmid. As shown in Fig. 7J, ectopic AR expression decreased the recruitment of Sirtuin 6 to PGC-1α. Sirtuin is deacetylase to downregulate PGC-1α acetylation, leading to increased transcriptional activity of PGC-1α [16]. Hence, we assessed the acetylation of PGC-1α by immunoprecipitation of PGC-1α with acetyl-lysine. As shown, overexpression of AR increased the expression of PGC-1α acetylation. To further identify the role of AR in PGC-1α acetylation, we isolated the nuclear fraction of DHT-treated cells. As shown in Fig. 7K, DHT induced AR location in nucleus and triggered PGC-1α acetylation. To clarify the inhibitory role of Sirtuin 6 in AR-induced PGC-1α acetylation, HKC-8 cells were transfected with Sirtuin 6 expressing plasmid and treated with DHT. As shown, DHT-induced PGC-1α acetylation was blocked by ectopic Sirtuin 6 (Fig. 7L). In addition, DHT-decreased PGC-1α expression was also reversed by ectopic Sirtuin 6 (Fig. 7L). We also isolated the nuclear fraction from IRI mice, and found that IRI injury induced AR localizing into nucleus and triggered PGC-1α acetylation (Fig. 7M). These results suggested that AR transcriptionally decreased Sirtuin 6, leading to PGC-1α acetylation and decreased activity of PGC-1α.

### Sirtuin 6 plays a key protective role against AR-induced tubular cell apoptosis and mitochondrial dysfunction

**Fig. 8:**
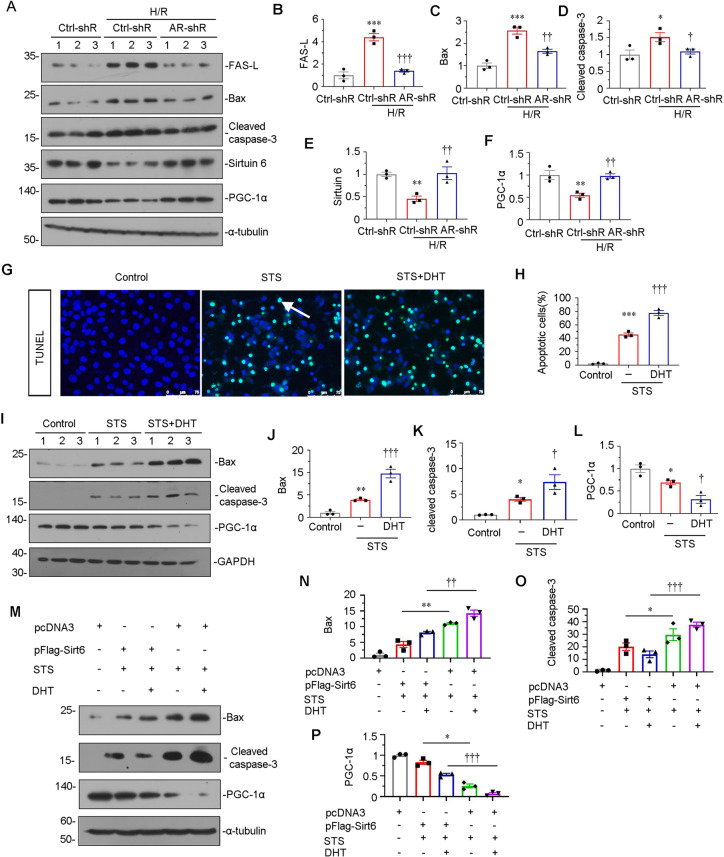
Sirtuin 6 plays a key role in AR-induced mitochondrial dysfunction and tubular cell apoptosis in vitro. **A**–**D** Representative western blot (**A**) and graphical representations of (**B**) Bax, (**C**) cleaved caspase-3 and (**D**) PGC-1α protein expression levels are shown. *P < 0.05, **P < 0.01 versus control group (*n* = 3); ^†^*P* < 0.05, ^†††^*P* < 0.001 versus STS group (*n* = 3). HKC-8 were treated with STS (1 μMol/L) alone or co-treated with DHT (10 μMol/L) for 10 h. **E**, **F** TUNEL assay (**E**) and quantitative data (**F**) showed the degree of cellular apoptosis in different groups, as indicated. Frozen kidney sections were stained by TUNEL staining. Arrows indicate positive staining. Scale bar, 75 μm. ***P < 0.001 versus control group (*n* = 3); ^†††^*P* < 0.001 versus STS group (*n* = 3). **G**–**L** Representative western blot (**G**) and graphical representations of (**H**) FAS-L, (**I**) Bax, (**J**) cleaved caspase-3, (**K**) Sirtuin 6 and (**L**) PGC-1α protein expression levels are shown. HKC-8 is transfected with control-shRNA or AR-shRNA before H/R injury. *P < 0.05, **P < 0.01, ***P < 0.001 versus control-shRNA group (*n* = 3); ^†^*P* < 0.05, ^††^*P* < 0.01, ^†††^*P* < 0.001 versus control-shRNA/H/R group (*n* = 3). **M**–**P** Representative western blot (**M**) and graphical representations of (**N**) Bax, (**O**) cleaved caspase-3 and (**P**) PGC-1α protein expression levels are shown. HKC-8 cells were transfected with Sirtuin 6 overexpression plasmid and treated with STS (1 μMol/L) or co-treated with DHT (10 μMol/L) for 6 h. *P < 0.05, **P < 0.01 (*n* = 3); ^††^*P* < 0.01, ^†††^*P* < 0.001 (*n* = 3). **Q** Androgen binds to the AR in cytoplasm, contributing to the translocation of AR from the cytoplasm into cell nucleus. The AR binds to Sirtuin 6 promoter and deregulates Sirtuin 6 expression. The decreased expression of Sirtuin 6 leads to decreased deacetylation of PGC-1α, contributing to decreased transcriptional activity of PGC-1α. It results in downregulation of mitochondrial biogenesis relative genes, such as TFAM, contributing to the imbalance of mitochondrial homeostasis, which further results in renal TECs apoptosis after AKI.

HKC-8 cells were knocked down AR expression and induced by hypoxia-reoxygenation (H/R) injury. As shown, H/R injury triggered apoptosis-related proteins FAS-L, Bax, cleaved caspase-3 expression, and downregulated the expression of Sirtuin 6 and PGC-1α, however, knockdown of AR largely inhibited these effects (Fig. 8A–F). To further explore the role of Sirtuin 6 in AR-induced cell apoptosis and mitochondrial dysfunction, HKC-8 cells were treated with staurosporin (STS), an inducer of apoptosis [46] and co-treated with DHT. TUNEL assay revealed co-treatment with DHT further elevated cell apoptosis (Fig. 8G, H). Furthermore, as shown in Fig. 8I–L, co-treatment with DHT further aggravated STS-induced Bax and cleaved caspase-3 expression and downregulated PGC-1α expression, suggesting the important role of AR in tubular cell apoptosis. HKC-8 cells were next transfected Sirtuin 6 expressing plasmid and treated with STS or DHT and STS. As shown in Fig. 8M–P, ectopic Sirtuin 6 significantly inhibited the expression of Bax and cleaved caspase-3 in STS or STS/DHT-treated cells. Moreover, overexpression of Sirtuin 6 also greatly preserved the expression of PGC-1α in STS or STS/DHT-treated cells.

**Fig. 9:**
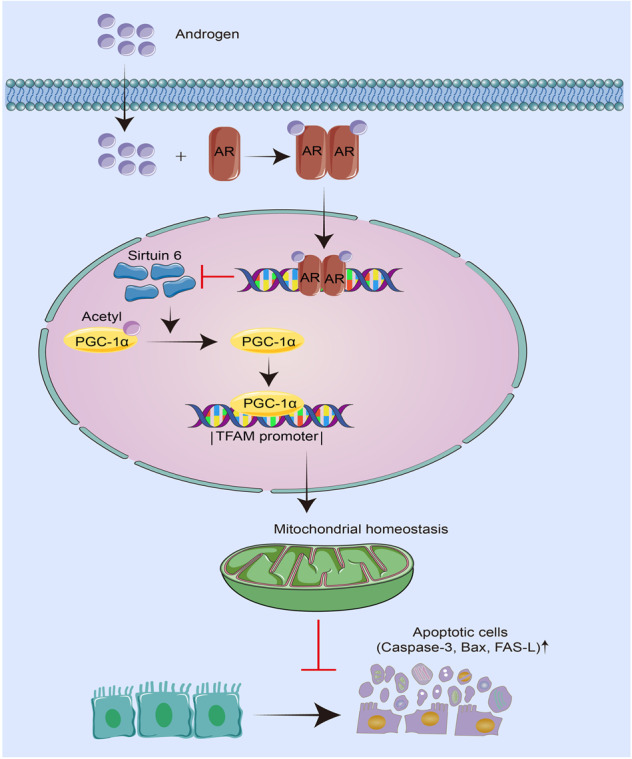
Androgen continually activates AR, which translocates into nucleus. As a transcriptional factor, AR binds with the promoter of Sirtuin 6 and inhibites its transcription and translation, resulting in PGC-1αacetylation and decreased activity of PGC-1α. This leads to downregulation of mitochondrial biogenesis-related genes such as mitochondrial transcription factor A (TFAM), which damages mitochondrial homeostasis and triggered cell apoptosis.

Collectively, our results suggest that the AR pathway plays an important role in the pathogenesis of renal tubular cell apoptosis (Fig. 9). In male AKI mice, androgen continually activates AR, which translocates into nucleus. As a transcriptional factor, AR binds with the promoter of Sirtuin 6, a deacetylase, and inhibits its transcription and translation. Downregulation of Sirtuin 6 results in PGC-1α acetylation and decreased activity of PGC-1α. This leads to downregulation of mitochondrial biogenesis-related genes such as mitochondrial transcription factor A (TFAM), which damages mitochondrial homeostasis and triggered cell apoptosis. This could be the underlying mechanism for gender differences in AKI injury.

## Discussion

AKI is at an increased morbidity, mortality and high costs [47]. Studies have found that gender difference exists in the incidence of AKI [4, 11]. The large epidemiologic and retrospective studies showed that female gender is a protective factor for ischemic AKI after non-cardiac surgery and nephrectomy induced-AKI, although this is still controversial [4, 10, 48–51]. However, in animal models, it has been widely reported that female mice shows high resistance to ischemic AKI [5, 52–56].

The mechanisms of gender dimorphism in AKI still remain unclear. Studies found that sex hormone may play an important role in this process, and the presence of testosterone is more important than the absence of estrogen [4]. However, the effects of testosterone in AKI are also controversial. Some studies have demonstrated the deleterious effects of testosterone. Park KM et al. found that testosterone increased inflammation and functional injury in IRI [5]. Another study found that orchidectomy attenuated post-ischemic oxidative stress and bilateral renal IRI [55]. Kyung PK et al. found that castration reduced the levels of IRI-induced tubular injury and macrophage infiltration while supplement of testosterone reversed this protective effect [56]. There are also opposite conclusions. A study found that low-dose of testosterone protects against renal bilateral IRI by increasing the ratio of IL-10/TNF-α, and attenuating T-cell infiltration [57]. It suggests that the role of androgen and this receptor in the sexual dimorphism in AKI required further studies. In our study, we found that male gender showed more severe kidney injury in IRI (Fig. 1) and rhabdomyolysis-induced AKI (Fig. 3). Ectopic knockdown of AR in male mice could rescue renal function (Fig. 5).

Renal tubular epithelial cells are at high energy requirement and with active metabolism. They are often the primary target of injury [12]. It has been shown that the mitochondrial dysfunction and following apoptosis in TECs play important roles in the development of AKI [1, 15, 21, 24]. Our study found that male mice exhibited more severe mitochondrial dysfunction and tubular cell apoptosis in both IRI (Figs. 1 and 2) and rhabdomyolysis-induced AKI models (Figs. 3 and 4). Ectopic knockdown of AR in male mice reversed these effects (Fig. 5). Moreover, the administration of DHT worsened STS-induced mitochondrial dysfunction and tubular cell apoptosis in vitro (Fig. 8). Ectopic knockdown of AR ameliorated H/R-induced mitochondrial dysfunction and cell apoptosis in vitro (Fig. 8). These results suggest that AR signaling pathway plays a key role in the susceptibility to AKI in male gender, and is associates with mitochondrial dysfunction and tubular cell apoptosis.

Sirtuins play critical roles in lifespan and metabolic adaptive responses to stress [31], also in cellular apoptosis [29, 58]. Studies have shown that a majority of Sirtuin members play significant roles in AKI. Reports showed that Sirtuin 1, 3 and 6 play important roles in alleviating kidney injury in AKI [31, 59], while Sirtuin 2 and 7 play opposite roles. Sirtuin 1 exerts renoprotective effects in AKI by eliminating renal ROS production and restoring mitochondrial biogenesis via activating PGC-1α [60]. Moreover, Sirtuin 3 protects against AKI by eliminating ROS, enhancing autophagy, regulating mitochondrial dynamics, inhibiting renal inflammation and improving fatty acid oxidation [35, 60, 61]. The study also showed Sirtuin 6 can attenuate inflammation and apoptosis in cisplatin-induced AKI via inhibiting ERK signaling [62], while silence of Sirtuin 2 and 7 attenuate renal inflammations in AKI [60, 63–65]. The role of Sirtuin 5 in AKI is controversial. It reports that Sirtuin 5 can inhibit tubular cell apoptosis in cisplatin-induced AKI via protecting mitochondria function and eliminating ROS [66, 67]. However, a study also found that knockout of Sirtuin 5 protects against AKI via enhancing tubular cell fatty acid oxidation [68]. In addition, the role of Sirtuin 4 in AKI remains unknown. Therefore, the role of all Sirtuins in AKI should be clarified in detail in the future.

In our study, we found that the expression of Sirtuins was downregulated in IRI model (Fig. 2). Furthermore, male mice exhibited loss of Sirtuins expression in IRI (Fig. 2) and rhabdomyolysis-induced AKI models (Fig. 4). Notably, Sirtuin 6 was preserved most in female mice (Figs. 2 and 4). Furthermore, ectopic expression of AR and DHT treatment decreased the expression of Sirtuin 6 at the most, among Sirtuin family (Fig. 7). A previous study showed that Sirtuin 6 can relieve AKI by promoting autophagy [59]. And it is also found that Sirtuin 6 can restore mitochondrial function and ameliorate apoptosis in podocytes [69, 70]. Consistently, our study found that overexpression of Sirtuin 6 alleviated DHT-induced mitochondrial dysfunction and tubular cell apoptosis in vitro (Fig. 8), suggesting that Sirtuin 6 is the key protective contributor against androgen-induced tubular apoptosis.

Some reports have shown that Sirtuin 1 and Sirtuin 3 could be regulated by androgen-mediated signaling pathway [36]. Moreover, Sirtuin 1 localized in nucleus can deacetylate and activate PGC-1α, further to enhance mitochondrial biogenesis [34]. Similarly, we found that AR can downregulate the transcription of Sirtuin 6, leading to decreased deacetylation of PGC-1α by Sirtuin 6 in nucleus (Fig. 7), which plays a key role in susceptibility of male mice in AKI.

In summary, our results showed AR-induced downregulation of Sirtuin 6 plays a key role in male gender susceptibility suffering from AKI. Sirtuin 6-mediated deacetylation of PGC-1α could protect against mitochondrial dysfunction and tubular cell apoptosis in AKI. Our study provided a new prospective to explain the underlying mechanisms of gender differences in AKI.

## Materials and methods

### Animal model

The animal experiments were approved by the Ethics Committee on Use and Care of Animals of Southern Medical University, Guangzhou, China. Eight-week-old male and female C57BL/6 mice, were purchased from Southern Medical University Animal Center (Guangzhou, China). Mice were randomized into different groups using random number table. 5 mice were included in each group to meet the minimum sample size requirement to perform one-way ANOVA analysis. Renal IRI was established by an established protocol [71]. In brief, bilateral renal pedicles were clipped for 30 minutes by microaneurysm clamps (item no.18051–35; Fine Science Tools, Cambridge, UK). During the ischemic period, mice body temperature was maintained at 38 °C by a temperature-controlled heating system. After the removal of clamps, reperfusion of the ischemic kidneys was visually confirmed. Mice were euthanized 24 h after IRI. Some male mice were injected with shRNA expression plasmid (control-shRNA or AR-shRNA) or overexpression plasmid (pcDNA3 or pFlag-Sirtuin 6 plasmid) by tail vein injection with a rapid and large volume plasmid solution as reported [71]. The sequence of AR-shRNA is in supplementary table 1. Serum and kidney tissues were collected for the following analyses. For rhabdomyolysis-induced AKI, mice were intramuscularly injected with 50% glycerol at the dose of 7.5 ml/kg. Mice were euthanized 3 days after glycerol intramuscular injection. Serum and kidney tissues were collected for the following analyses.

### Determination of serum creatinine (Scr) and blood urea nitrogen (BUN)

The levels of Scr and BUN were measured with an AU480 Automatic biochemical analyzer (Beckman Coulter, Brea, CA). Scr and serum BUN were expressed as milligrams per 100 ml.

### Cell culture and treatment

Human proximal tubular epithelial cells (HKC-8) were given by Dr. L. Racusen (Johns Hopkins University, Baltimore, MD, USA). Cell culture was performed as previously described [37]. The empty vector (pcDNA3), or Sirtuin 6 overexpression plasmid (pFlag-Sirt6) or control-shRNA or AR-shRNA was transfected by Lipofectamine 2000 reagent (Invitrogen, Grand Island, NY, USA). Some cells were also treated with dihydrotestosterone (DHT) (ID0310-2; Solarbio) (10 μmol/L) or staurosporine (STS) (HY-15141; MCE) (1 μmol/L).

### Model of hypoxia/reoxygenation (H/R) in vitro

The H/R was performed with routine protocol. H/R injury was induced in normal HKC-8 by incubation in basal culture medium in a 1% O_2_ environment for 24 h followed by reoxygenation with normal O_2_ for 8 h.

### Western blot analysis

Protein expression was analyzed by Western blot analysis. The primary antibodies were as follows: anti-AR (BA34204; Boster), anti-Bax (SC-7480; Santa Cruz Biotechnology), anti-caspase-3 (SC-65497; Santa Cruz Biotechnology), anti-Flag-tag (M185-3S; MBL), anti-FAS-L (SC-19681; Santa Cruz Biotechnology), anti-GAPDH (RM2001; Ray Antibody Biotech), anti-KIM-1 (BA3537; Boster), anti-PGC-1α (ab54481; Abcam), anti-Sirtuin 1 (PB0173; Boster), anti-Sirtuin 2 (PB9160; Boster), anti-Sirtuin 3 (PB0175; Boster), anti-Sirtuin 4 (A15800; ABclonal), anti-Sirtuin 5 (ab108968; Abcam), anti-Sirtuin 6 (PB0375; Boster), anti-Sirtuin 7 (PB0376; Boster), anti-TOMM20 (ab186735; Abcam), anti-α-tubulin (RM2007; Ray Antibody Biotech, Beijing, China), anti-β-actin (RM2001; Beijing Ray Antibody Biotech).

### Nuclear fraction isolation

Nuclear fractions were separated with a commercial kit (BestBio, Shanghai, China), according to the manufacturer’s instruction.

### Histology and immunohistochemical staining

Paraffin kidney sections (3 μm) were stained with periodic acid–Schiff (PAS) reagent by standard protocol. Immunohistochemical staining was performed with routine protocol [37]. Antibodies used were as follows: anti-caspase-3 (SC-65497; Santa Cruz Biotechnology), anti-KIM-1 (BA3537; Boster), anti-Sirtuin 6 (PB0375; Boster).

### Immunofluorescence staining

Immunofluorescence staining was performed with routine protocol [72]. Frozen kidney sections (3 μm) were fixed with 4% paraformaldehyde. All primary antibodies used were as follows: anti-TOMM20 (ab186735; Abcam), anti-Sirtuin 6 (PB0375; Boster), anti-PGC-1α (ab54481; Abcam). Then the slides were stained with Cy3-or Cy2-conjugated secondary antibodies (Jackson Immuno-Research Laboratories, West Grove, PA, United States). Nuclei were treated with DAPI (Sigma-Aldrich) under the manufacturer’s specifications.

### TUNEL assay

Frozen kidney sections (3 μm) or cells cultured on coverslips were assessed by TUNEL assay (KGA7073; Keygen), according to the manufacturer’s instruction.

### Reverse transcriptase and real-time quantitative PCR

Total RNA was extracted using a TRIzol RNA isolation system (Life Technologies, Grand Island, NY). Real-time quantitative PCR was performed on an ABI PRISM 7000 Sequence Detection System (Applied Bio-systems, Foster City, CA). The sequences of the primer pairs used in quantitative real-time PCR are described in Supplementary Table 2.

### Immunoprecipitation (IP)

IP experiments were performed with previous protocol [73]. Briefly, the cell lysates were immunoprecipitated overnight with anti-PGC-1α (SC-518038; Santa Cruz Biotechnology) and protein A/G plus agarose (sc-2003; Santa Cruz Biotechnology) at 4 °C. The precipitated complexes were washed three times with lysis buffer and boiled for 10 min in SDS sample buffer. And the next progression is similar to western blot. The primary antibodies of following western blot were as follows: anti-Sirtuin 6 (PB0375; Boster), anti-acetyl-lysine (9441 s; Cell Signaling Technology).

### Chromatin immunoprecipitation (ChIP)

HKC‐8 cells were incubated with DHT for 24 h. Cells were fixed with 4% formaldehyde for 10 min at room temperature for protein‐DNA crosslinking. Cell lysates were obtained and the ChIP assay was performed using the Simple ChIP Plus (Magnetic Bead) Kit (Cell Signaling, Cat. 9005). The antibody against AR (BA34204; Boster), normal rabbit IgG and H3 was added and incubated overnight at 4 °C, followed by incubation with protein A‐agarose for 1 h. After washing out the precipitate, purified DNA was used as a template for PCR. The sequences of human Sirtuin 6 primers were as follows: forward 5′‐CCACGATCTTCCCTATCATCA‐3′ and reverse 5′‐AGCATGAAACTCCGTCTCAAA‐3′.

### Statistical analyses

Statistical analysis was performed by researcher who was blinded. All data were expressed as mean ± SEM. Statistical analysis was carried out by SPSS 25.0 (SPSS Inc, Chicago, IL, USA). Unpaired *t* test was used to compare two groups. Comparison between groups was made via one-way ANOVA analysis of variance followed by the Least Significant Difference when the variance between groups was homogeneous, or the Dunnett T3 test when the variance between groups was not homogeneous. Fisher’s exact test was used to compare two rate. A *P* value < 0.05 was considered as significant. Quantification of positive staining was assessed by two researchers who were blinded through Image Pro Plus software.

## Supplementary information

Supplementary data

wesrtern blot

## Data Availability

The data used to support the findings of this study are available from the corresponding author upon request.

## References

[1] Linkermann A, Chen G, Dong G, Kunzendorf U, Krautwald S, Dong Z (2014). Regulated cell death in AKI. J Am Soc Nephrol.

[2] Guo C, Dong G, Liang X, Dong Z (2019). Epigenetic regulation in AKI and kidney repair: mechanisms and therapeutic implications. Nat Rev Nephrol.

[3] Huang J, Kong Y, Xie C, Zhou L (2021). Stem/progenitor cell in kidney: characteristics, homing, coordination, and maintenance. Stem Cell Res Ther.

[4] Kim NY, Lee HS, Park JH, Jeon S, Oh C, Kim SY (2021). Influence of age on gender-related differences in acute kidney injury after minimally invasive radical or partial nephrectomy. Surgical Endosc.

[5] Park KM, Kim JI, Ahn Y, Bonventre AJ, Bonventre JV (2004). Testosterone is responsible for enhanced susceptibility of males to ischemic renal injury. J Biol Chem.

[6] Hester J, Ventetuolo C, Lahm T (2019). Sex, gender, and sex hormones in pulmonary hypertension and right ventricular failure. Compr Physiol.

[7] Ngo ST, Steyn FJ, McCombe PA (2014). Gender differences in autoimmune disease. Front Neuroendocrinol.

[8] Picillo M, Nicoletti A, Fetoni V, Garavaglia B, Barone P, Pellecchia MT (2017). The relevance of gender in Parkinson’s disease: a review. J Neurol.

[9] Podcasy JL, Epperson CN (2016). Considering sex and gender in Alzheimer disease and other dementias. Dialogues Clin Neurosci.

[10] Hutchens MP, Dunlap J, Hurn PD, Jarnberg PO (2008). Renal ischemia: does sex matter. Anesthesia Analgesia.

[11] Metcalfe PD, Meldrum KK (2006). Sex differences and the role of sex steroids in renal injury. J Urol.

[12] Zhou D, Liu Y (2016). Renal fibrosis in 2015: understanding the mechanisms of kidney fibrosis. Nat Rev Nephrol.

[13] Chen S, Zhang M, Li J, Huang J, Zhou S, Hou X (2022). β-catenin-controlled tubular cell-derived exosomes play a key role in fibroblast activation via the OPN-CD44 axis. J Extracell Vesicles.

[14] Forbes JM, Thorburn DR (2018). Mitochondrial dysfunction in diabetic kidney disease. Nat Rev Nephrol.

[15] Zhang X, Agborbesong E, Li X. The Role of Mitochondria in Acute Kidney Injury and Chronic Kidney Disease and Its Therapeutic Potential. Int J Mol Sci. 2021;22. 10.3390/ijms222011253. 10.3390/ijms222011253 PMC8537003 34681922

[16] Liang Z, Currais A, Soriano-Castell D, Schubert D, Maher P (2021). Natural products targeting mitochondria: emerging therapeutics for age-associated neurological disorders. Pharm Ther.

[17] Pickles S, Vigié P, Youle RJ (2018). Mitophagy and Quality Control Mechanisms in Mitochondrial Maintenance. Curr Biol: CB.

[18] Kang HM, Ahn SH, Choi P, Ko YA, Han SH, Chinga F (2015). Defective fatty acid oxidation in renal tubular epithelial cells has a key role in kidney fibrosis development. Nat Med.

[19] Dan Dunn J, Alvarez LA, Zhang X, Soldati T (2015). Reactive oxygen species and mitochondria: a nexus of cellular homeostasis. Redox Biol.

[20] Zhao M, Wang Y, Li L, Liu S, Wang C, Yuan Y (2021). Mitochondrial ROS promote mitochondrial dysfunction and inflammation in ischemic acute kidney injury by disrupting TFAM-mediated mtDNA maintenance. Theranostics.

[21] Yang Q, Ren GL, Wei B, Jin J, Huang XR, Shao W (2019). Conditional knockout of TGF-βRII /Smad2 signals protects against acute renal injury by alleviating cell necroptosis, apoptosis and inflammation. Theranostics.

[22] Tower J (2015). Programmed cell death in aging. Ageing Res Rev.

[23] Maiuri MC, Zalckvar E, Kimchi A, Kroemer G (2007). Self-eating and self-killing: crosstalk between autophagy and apoptosis. Nat Rev Mol Cell Biol.

[24] Liu H, Wang L, Weng X, Chen H, Du Y, Diao C (2019). Inhibition of Brd4 alleviates renal ischemia/reperfusion injury-induced apoptosis and endoplasmic reticulum stress by blocking FoxO4-mediated oxidative stress. Redox Biol.

[25] Lai JJ, Chang P, Lai KP, Chen L, Chang C (2012). The role of androgen and androgen receptor in skin-related disorders. Arch Dermatol. Res.

[26] Culig Z, Santer FR (2014). Androgen receptor signaling in prostate cancer. Cancer Metastasis Rev.

[27] Lin Y, Kokontis J, Tang F, Godfrey B, Liao S, Lin A (2006). Androgen and its receptor promote Bax-mediated apoptosis. Mol Cell Biol.

[28] Kher A, Meldrum KK, Wang M, Tsai BM, Pitcher JM, Meldrum DR (2005). Cellular and molecular mechanisms of sex differences in renal ischemia-reperfusion injury. Cardiovasc. Res.

[29] Carafa V, Rotili D, Forgione M, Cuomo F, Serretiello E, Hailu GS (2016). Sirtuin functions and modulation: from chemistry to the clinic. Clin Epigenet.

[30] Peng L, Qian M, Liu Z, Tang X, Sun J, Jiang Y (2020). Deacetylase-independent function of SIRT6 couples GATA4 transcription factor and epigenetic activation against cardiomyocyte apoptosis. Nucleic Acids Res.

[31] Morigi M, Perico L, Benigni A (2018). Sirtuins in renal health and disease. J Am Soc Nephrol.

[32] Wątroba M, Dudek I, Skoda M, Stangret A, Rzodkiewicz P, Szukiewicz D (2017). Sirtuins, epigenetics and longevity. Ageing Res Rev.

[33] Miao J, Liu J, Niu J, Zhang Y, Shen W, Luo C (2019). Wnt/β-catenin/RAS signaling mediates age-related renal fibrosis and is associated with mitochondrial dysfunction. Aging Cell.

[34] Rodgers JT, Lerin C, Haas W, Gygi SP, Spiegelman BM, Puigserver P (2005). Nutrient control of glucose homeostasis through a complex of PGC-1alpha and SIRT1. Nature.

[35] Shen H, Holliday M, Sheikh-Hamad D, Li Q, Tong Q, Hamad CD (2021). Sirtuin-3 mediates sex differences in kidney ischemia-reperfusion injury. Transl Res: J Lab Clin Med.

[36] Moore RL, Dai Y, Faller DV (2012). Sirtuin 1 (SIRT1) and steroid hormone receptor activity in cancer. J Endocrinol.

[37] Zhou L, Li Y, Hao S, Zhou D, Tan RJ, Nie J (2015). Multiple genes of the renin-angiotensin system are novel targets of Wnt/β-catenin signaling. J Am Soc Nephrol.

[38] Han WK, Bailly V, Abichandani R, Thadhani R, Bonventre JV (2002). Kidney Injury Molecule-1 (KIM-1): a novel biomarker for human renal proximal tubule injury. Kidney Int.

[39] Ke H, Wang X, Zhou Z, Ai W, Wu Z, Zhang Y (2021). Effect of weimaining on apoptosis and Caspase-3 expression in a breast cancer mouse model. J Ethnopharmacol.

[40] Mirzayans R, Murray D. Do TUNEL and other apoptosis assays detect cell death in preclinical studies? Int J Mol Sci. 2020;21. 10.3390/ijms21239090. 10.3390/ijms21239090 PMC7730366 33260475

[41] Mitsiades N, Poulaki V, Mitsiades CS, Koutras DA, Chrousos GP (2001). Apoptosis induced by FasL and TRAIL/Apo2L in the pathogenesis of thyroid diseases. Trends Endocrinol Metab.

[42] Dong L, Vaux DL (2020). Glucocorticoids can induce BIM to trigger apoptosis in the absence of BAX and BAK1. Cell Death Dis.

[43] Hentzen NB, Mogaki R, Otake S, Okuro K, Aida T (2020). Intracellular photoactivation of caspase-3 by molecular glues for spatiotemporal apoptosis induction. J Am Chem Soc.

[44] Ma H, Guo X, Cui S, Wu Y, Zhang Y, Shen X (2022). Dephosphorylation of AMP-activated protein kinase exacerbates ischemia/reperfusion-induced acute kidney injury via mitochondrial dysfunction. Kidney Int.

[45] Miao J, Huang J, Luo C, Ye H, Ling X, Wu Q (2021). Klotho retards renal fibrosis through targeting mitochondrial dysfunction and cellular senescence in renal tubular cells. Physiol. Rep..

[46] Belmokhtar CA, Hillion J, Ségal-Bendirdjian E (2001). Staurosporine induces apoptosis through both caspase-dependent and caspase-independent mechanisms. Oncogene.

[47] Hoste EAJ, Kellum JA, Selby NM, Zarbock A, Palevsky PM, Bagshaw SM (2018). Global epidemiology and outcomes of acute kidney injury. Nat Rev Nephrol.

[48] Neugarten J, Sandilya S, Singh B, Golestaneh L (2016). Sex and the risk of AKI following cardio-thoracic surgery: a meta-analysis. Clin J Am Soc Nephrol.

[49] Brown JR, Cochran RP, Leavitt BJ, Dacey LJ, Ross CS, MacKenzie TA (2007). Multivariable prediction of renal insufficiency developing after cardiac surgery. Circulation.

[50] Ryckwaert F, Boccara G, Frappier JM, Colson PH (2002). Incidence, risk factors, and prognosis of a moderate increase in plasma creatinine early after cardiac surgery. Crit Care Med.

[51] Arora P, Rajagopalam S, Ranjan R, Kolli H, Singh M, Venuto R (2008). Preoperative use of angiotensin-converting enzyme inhibitors/angiotensin receptor blockers is associated with increased risk for acute kidney injury after cardiovascular surgery. Clin J Am Soc Nephrology.

[52] Tanaka R, Tsutsui H, Ohkita M, Takaoka M, Yukimura T, Matsumura Y (2013). Sex differences in ischemia/reperfusion-induced acute kidney injury are dependent on the renal sympathetic nervous system. Eur J Pharm.

[53] Hosszu A, Antal Z, Veres-Szekely A, Lenart L, Balogh DB, Szkibinszkij E (2018). The role of Sigma-1 receptor in sex-specific heat shock response in an experimental rat model of renal ischaemia/reperfusion injury. Transpl Int.

[54] Rusai K, Prókai A, Szebeni B, Mészáros K, Fekete A, Szalay B (2011). Gender differences in serum and glucocorticoid regulated kinase-1 (SGK-1) expression during renal ischemia/reperfusion injury. Cell Physiol Biochem.

[55] Kim J, Kil IS, Seok YM, Yang ES, Kim DK, Lim DG (2006). Orchiectomy attenuates post-ischemic oxidative stress and ischemia/reperfusion injury in mice. A role manganese superoxide dismutase. J Biol Chem.

[56] Kang KP, Lee JE, Lee AS, Jung YJ, Kim D, Lee S (2014). Effect of gender differences on the regulation of renal ischemia-reperfusion-induced inflammation in mice. Mol Med Rep..

[57] Patil CN, Wallace K, LaMarca BD, Moulana M, Lopez-Ruiz A, Soljancic A (2016). Low-dose testosterone protects against renal ischemia-reperfusion injury by increasing renal IL-10-to-TNF-α ratio and attenuating T-cell infiltration. Am J Physiol Ren Physiol.

[58] Bai X, Yao L, Ma X, Xu X (2018). Small Molecules as SIRT Modulators. Mini Rev Med. Chem.

[59] Zhang Y, Wang L, Meng L, Cao G, Wu Y (2019). Sirtuin 6 overexpression relieves sepsis-induced acute kidney injury by promoting autophagy. Cell Cycle.

[60] Huang C, Jiang S, Gao S, Wang Y, Cai X, Fang J (2022). Sirtuins: Research advances on the therapeutic role in acute kidney injury. Phytomedicine.

[61] Li M, Li CM, Ye ZC, Huang J, Li Y, Lai W (2020). Sirt3 modulates fatty acid oxidation and attenuates cisplatin-induced AKI in mice. J Cell Mol Med.

[62] Li Z, Xu K, Zhang N, Amador G, Wang Y, Zhao S (2018). Overexpressed SIRT6 attenuates cisplatin-induced acute kidney injury by inhibiting ERK1/2 signaling. Kidney Int.

[63] Jung YJ, Lee AS, Nguyen-Thanh T, Kim D, Kang KP, Lee S (2015). SIRT2 regulates LPS-induced renal tubular CXCL2 and CCL2 expression. J Am Soc Nephrol.

[64] Sánchez-Navarro A, Martínez-Rojas M, Albarrán-Godinez A, Pérez-Villalva R, Auwerx J, de la Cruz A, et al. Sirtuin 7 deficiency reduces inflammation and tubular damage induced by an episode of acute kidney injury. Int J Mol Sci. 2022;23. 10.3390/ijms23052573. 10.3390/ijms23052573 PMC8910458 35269715

[65] Miyasato Y, Yoshizawa T, Sato Y, Nakagawa T, Miyasato Y, Kakizoe Y (2018). Sirtuin 7 deficiency ameliorates cisplatin-induced acute kidney injury through regulation of the inflammatory response. Sci Rep..

[66] Li W, Yang Y, Li Y, Zhao Y, Jiang H (2019). Sirt5 attenuates cisplatin-induced acute kidney injury through regulation of Nrf2/HO-1 and Bcl-2. Biomed Res Int.

[67] Haschler TN, Horsley H, Balys M, Anderson G, Taanman JW, Unwin RJ (2021). Sirtuin 5 depletion impairs mitochondrial function in human proximal tubular epithelial cells. Sci Rep..

[68] Chiba T, Peasley KD, Cargill KR, Maringer KV, Bharathi SS, Mukherjee E (2019). Sirtuin 5 regulates proximal tubule fatty acid oxidation to protect against AKI. J Am Soc Nephrol.

[69] Fan Y, Yang Q, Yang Y, Gao Z, Ma Y, Zhang L (2019). Sirt6 suppresses high glucose-induced mitochondrial dysfunction and apoptosis in podocytes through AMPK activation. Int J Biol Sci.

[70] Fan Y, Cheng J, Yang Q, Feng J, Hu J, Ren Z (2021). Sirt6-mediated Nrf2/HO-1 activation alleviates angiotensin II-induced DNA DSBs and apoptosis in podocytes. Food Funct.

[71] Luo C, Zhou S, Zhou Z, Liu Y, Yang L, Liu J (2018). Wnt9a promotes renal fibrosis by accelerating cellular senescence in tubular epithelial cells. J Am Soc Nephrol.

[72] Meng P, Huang J, Ling X, Zhou S, Wei J, Zhu M (2022). CXC chemokine receptor 2 accelerates tubular cell senescence and renal fibrosis via β-catenin-induced mitochondrial dysfunction. Front Cell Dev. Biol.

[73] Zhou L, Li Y, He W, Zhou D, Tan RJ, Nie J (2015). Mutual antagonism of Wilms’ tumor 1 and β-catenin dictates podocyte health and disease. J Am Soc Nephrol.

