# Supplementary material for: Sirtuin 6 is a key contributor to gender differences in acute kidney injury
Source: Cell Death Discov. 2023 Apr 25;9:134. doi: 10.1038/s41420-023-01432-y (PMC10130034; doi:10.1038/s41420-023-01432-y)
Supplement: Supplementary file 1 — Supplementary data [file 41420_2023_1432_MOESM1_ESM.docx]

**
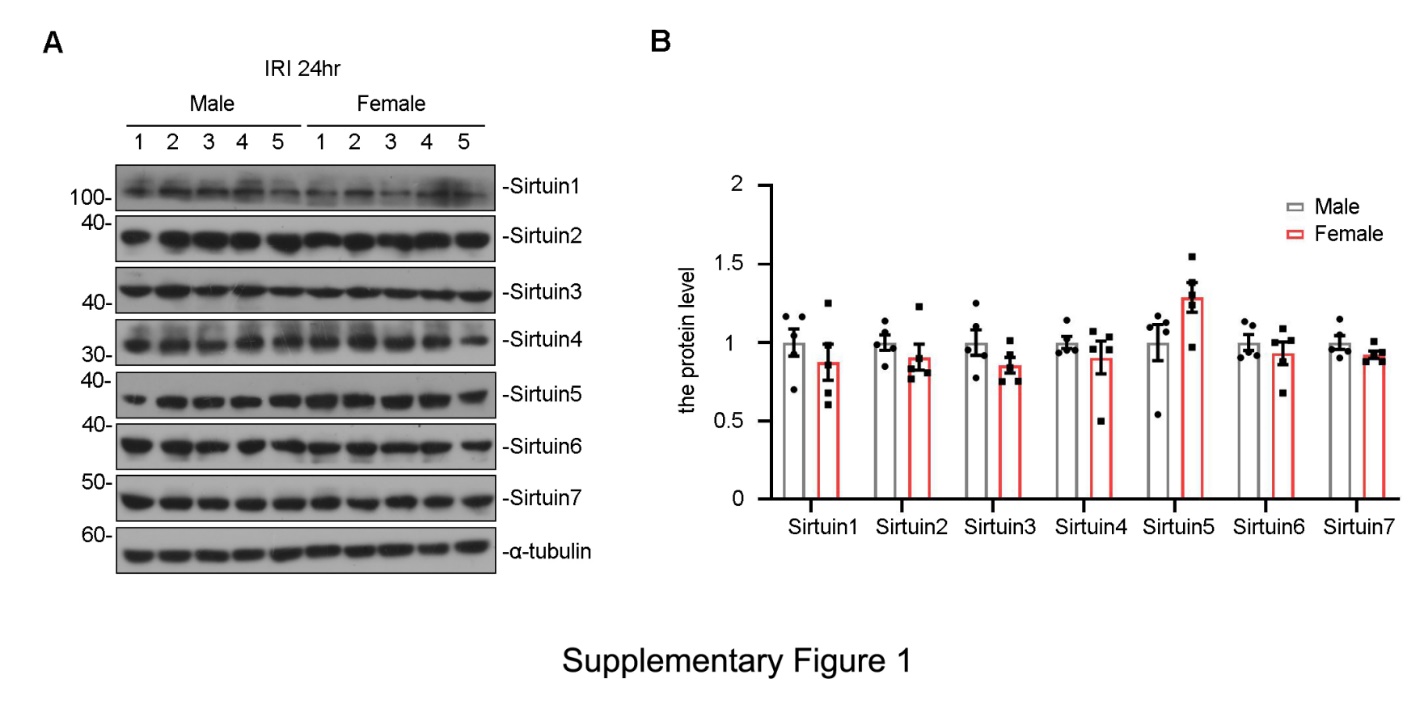
Supplementary Figure S1**

**Supplementary Figure S1. The expression of Sirtuins in male and female control mice.**

**(A-B)** Representative western blot (A) and graphical representations of (B) Sirtuin 1-7 protein expression levels are shown. n=5.

***Supplementary table 1. Nucleotide sequence of AR-shRNA***

Sequence (5′–3′)

| Sense:  CACCGCTACTCTTCAGCATTATTCCTTCAAGAGAGGAATAATGCTGAAGAGTAGCTTTTTTG |
| --- |
| Anti-sense: GATCCAAAAAAGCTACTCTTCAGCATTATTCCTCTCTTGAAGGAATAATGCTGAAGAGTAGC |

***Supplementary table 2. Nucleotide sequence of the primer used for qRT-PCR***

| Primer Sequence (5' to 3')  Gene Forward Reverse |
| --- |
| Mouse:  β-actin 5'- CAGCTGAGAGGGAAATCGTG -3' 5'- CGTTGCCAATAGTGATGACC -3'  Sirtuin 1 5'- CTTTCAGAACCACCAAAGCGG -3' 5'- CAAGGCGAGCATAGATACCGT -3'  Sirtuin 2 5'- CAGACTTCTCCAAGGTGGACC-3' 5'- ACGTCCCTGTAAGCCTTCTTG -3'  Sirtuin 3 5'- TGTAACAGCTACATGCACGGT -3' 5'- AGATCTGCCAAAGCGAAGTCA -3'  Sirtuin 4 5'- TCCGGCATCCCAGACTACA -3' 5'- AGAATTGAGGCCAGCCCAC -3'  Sirtuin 5 5'- ATCCCAGTCGACAAACTTCCC -3' 5'- CCACCACTAGACACAGGTCAC -3  Sirtuin 6 5'- AGCAGGGTTGTCGCCTTAC -3' 5'- GCTGGAGGACTGCCACATT -3  Sirtuin 7 5'- CTACATCGTGAACCTGCAGTG -3' 5'- CAGGCCCAGTTCATTCATGAG -3'  Human  β-actin 5'--CTCACCATGGATGATGATATCGC-3' 5'-AGGAATCCTTCTGACCCATGC-3'  Sirtuin 1 5'-CGGTTCCTACTGCGCGA-3' 5'-ATGAAACAGACACCTATCCGTGG-3'  Sirtuin 2 5'-CCTCCTTGCAGGGACGTGG-3' 5'-GACTTCTTGGGGGAAGCTGAA-3'  Sirtuin 3 5'-ACATTCGGGCTGACGTGATG-3' 5'-TTGATGAGCAGTCGGGGAAC-3'  Sirtuin 4 5'-CCACTGTGGGGTGTGAAGTGT-3' 5'-CGGTCAGTGCGGGCATAAAG-3'  Sirtuin 5 5'-CGTGGTCATCACCCAGAACA-3' 5'-GCCTCTTCACACCCTTTTCCT-3'  Sirtuin 6 5'-TCGACACCACCTTTGAGAGC-3' 5'-CGGACGTACTGCGTCTTACA-3'  Sirtuin 7 5'-CCGTCCGGAACGCCAAATA-3' 5'-TCAGGTCGGCAGCACTAAC-3' |
