# Supplementary material for: Sirtuin 6 is a key contributor to gender differences in acute kidney injury
Source: Cell Death Discov. 2023 Apr 25;9:134. doi: 10.1038/s41420-023-01432-y (PMC10130034; doi:10.1038/s41420-023-01432-y)

Full unedited gels for figure 1E

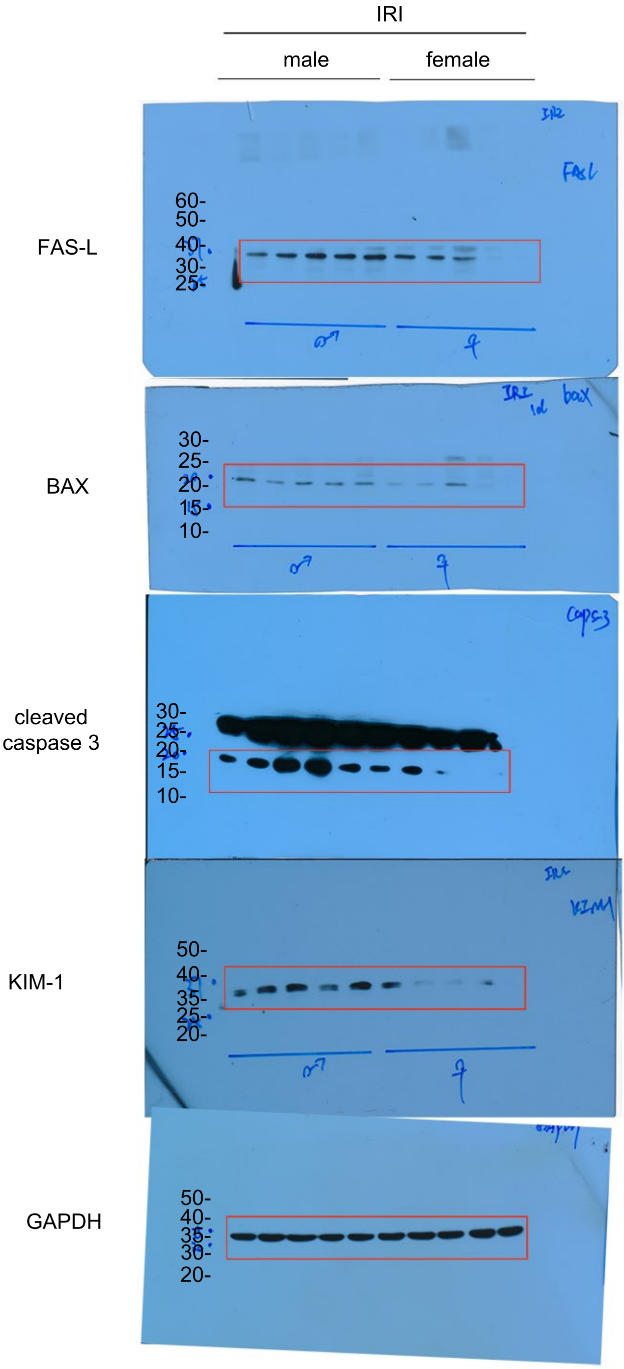

Full unedited gels for Figure 2B

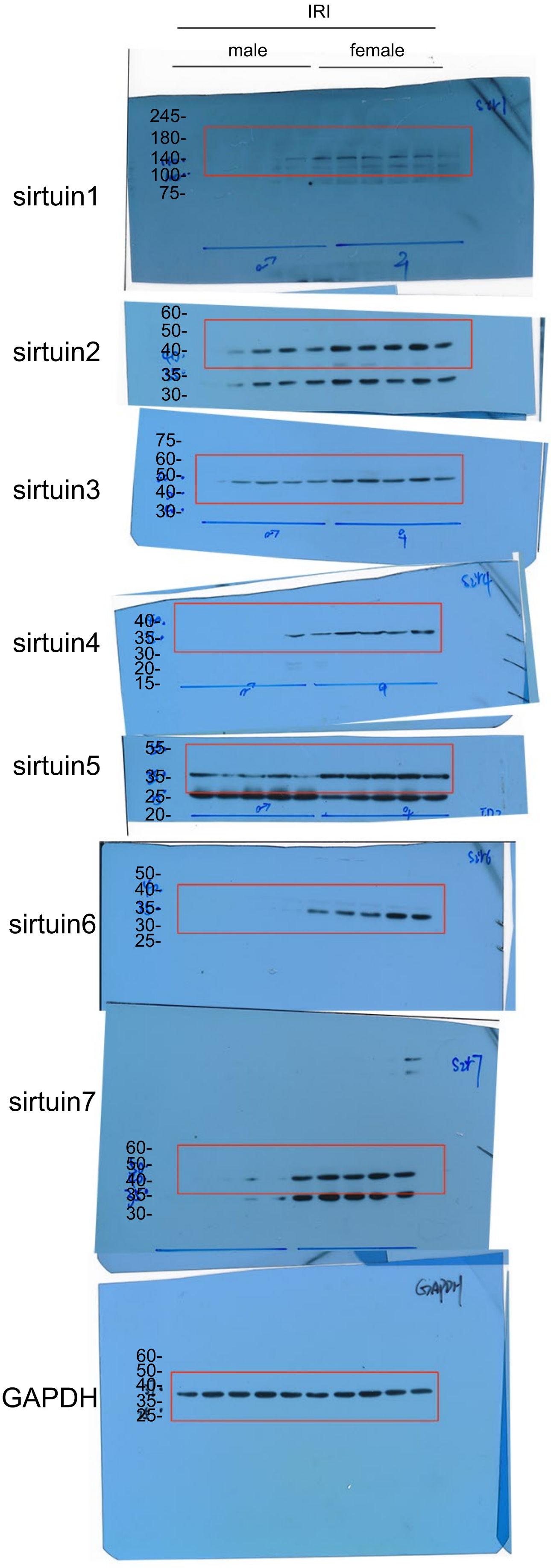

Full unedited gels for Figure 2F

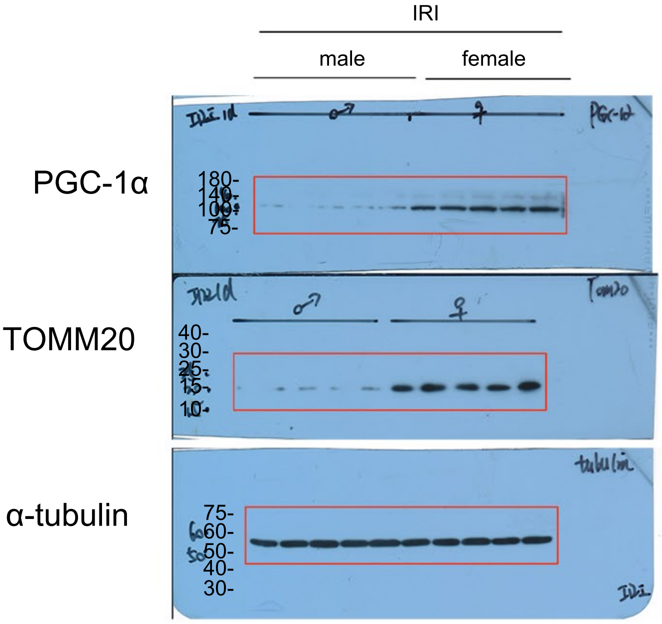

Full unedited gels for Figure 3F

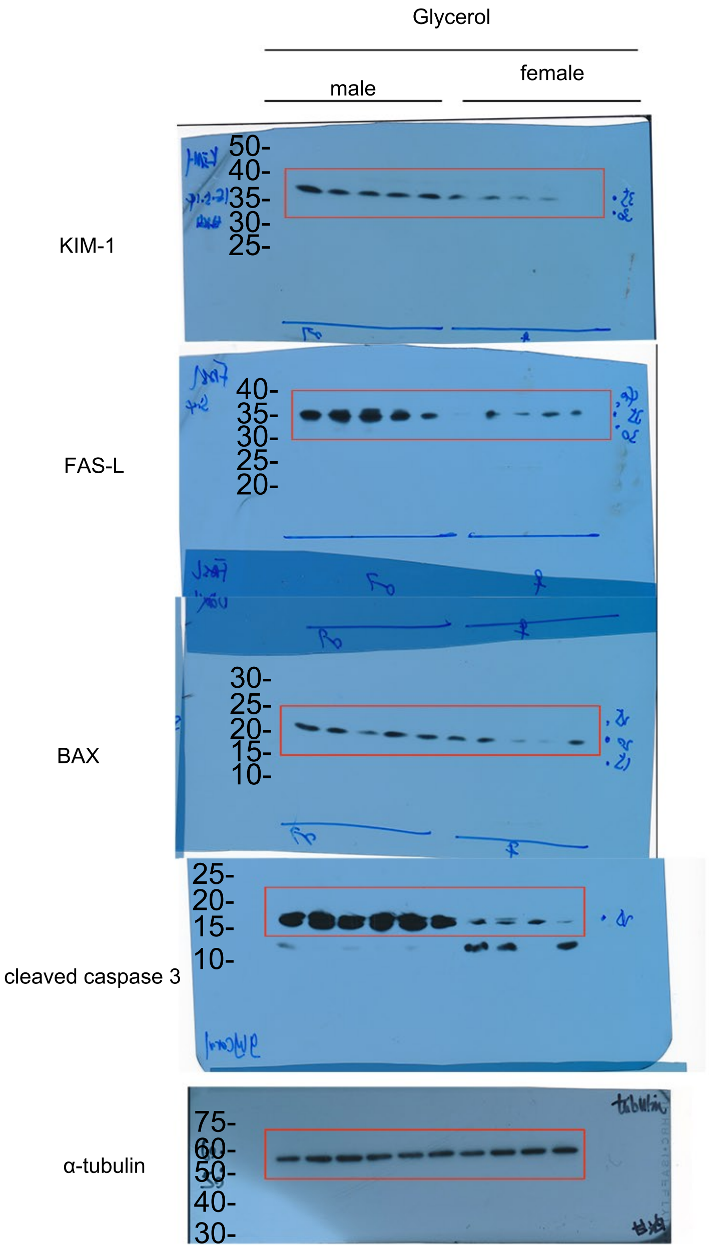

Full unedited gels for Figure 4A

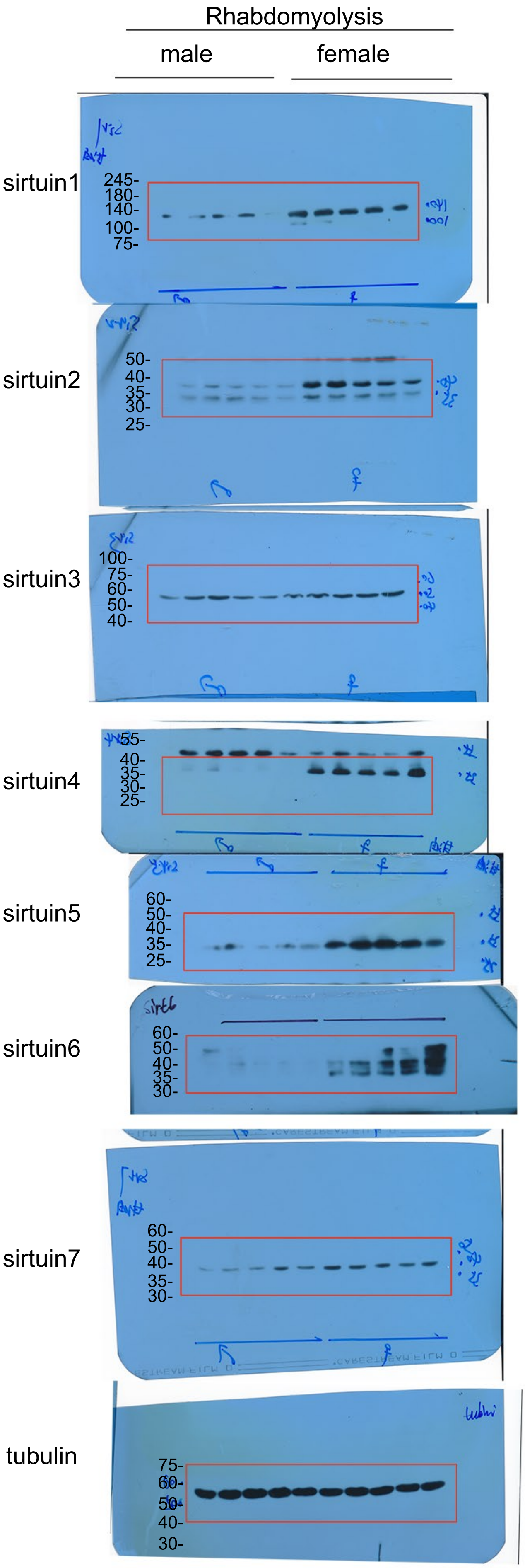

Full unedited gels for Figure 4E

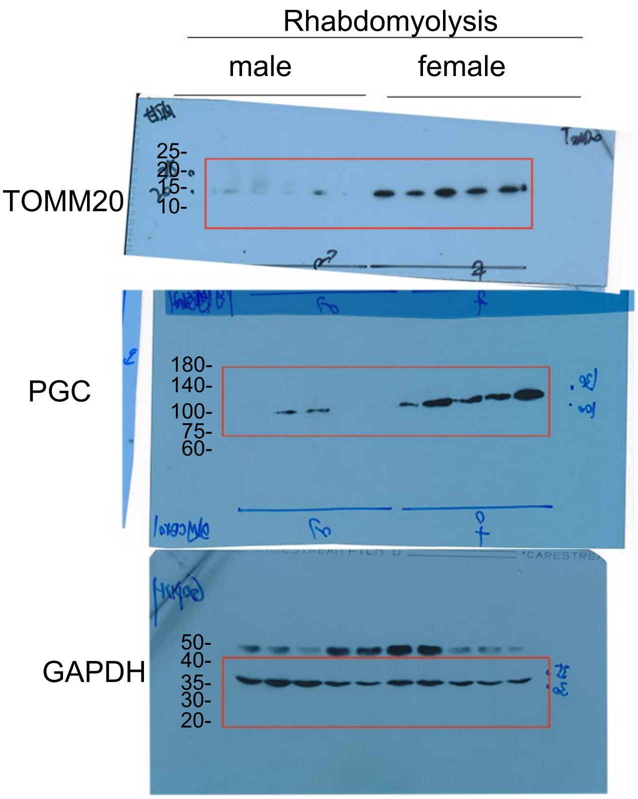

Full unedited gels for Figure 5D

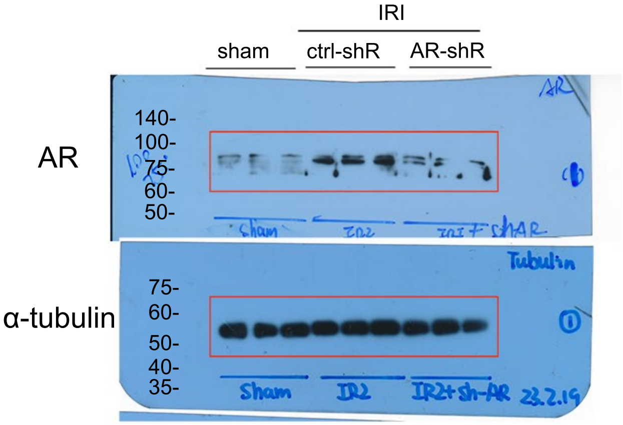

Full unedited gels for Figure 5J

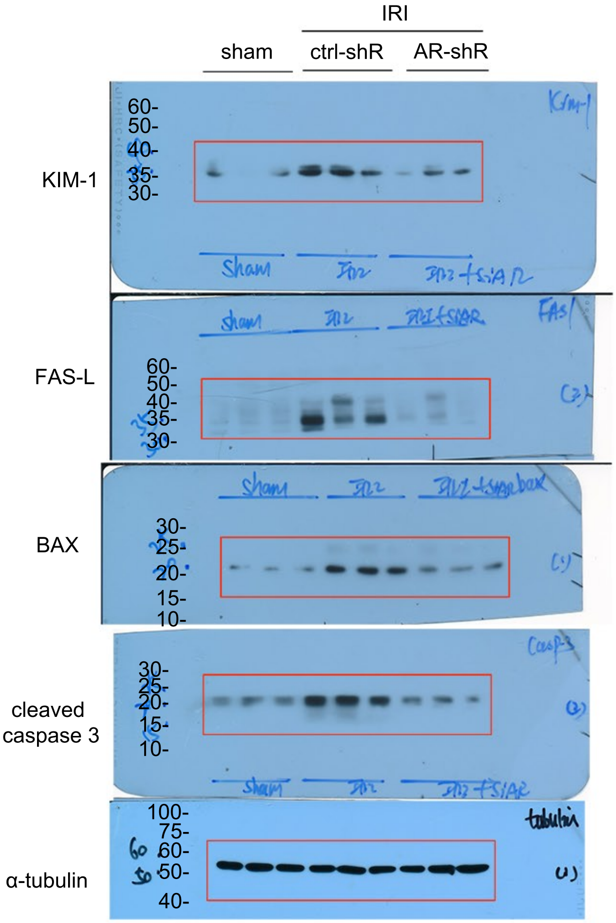

Full unedited gels for Figure 5Q

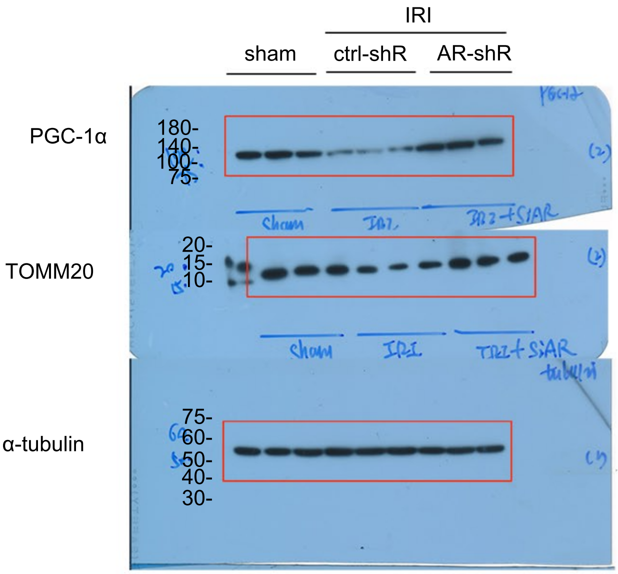

Full unedited gels for Figure 5F

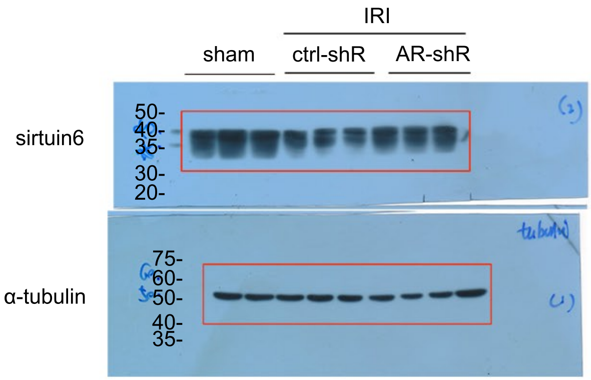

Full unedited gels for Figure 6C

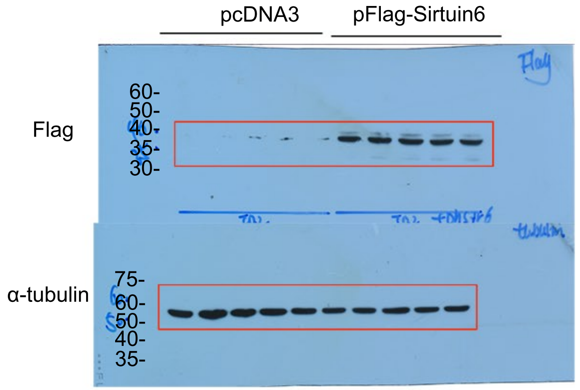

Full unedited gels for Figure 6E

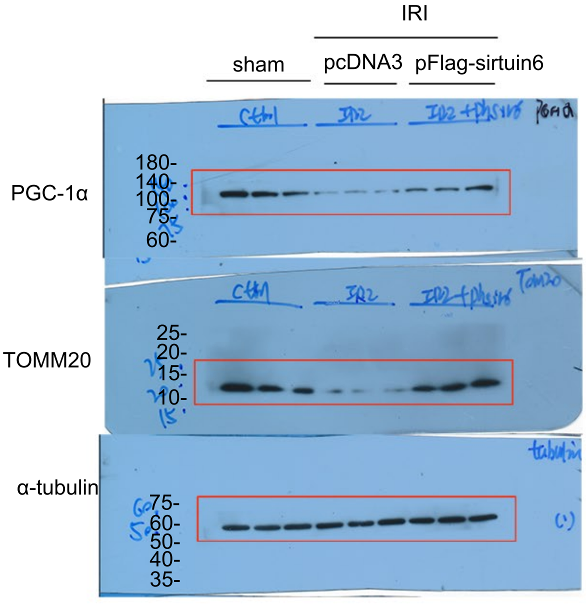

Full unedited gels for Figure 6I

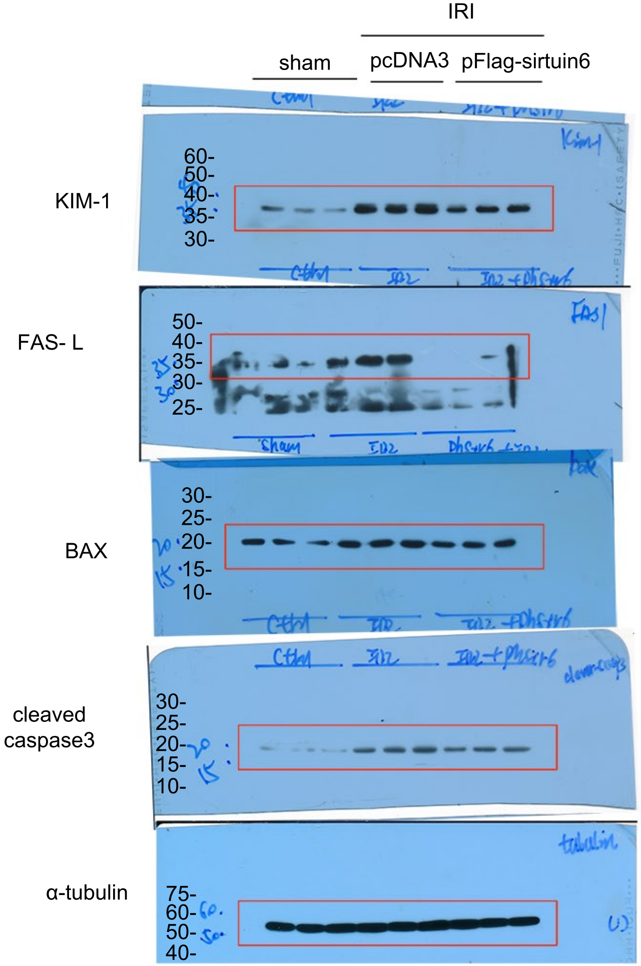

Full unedited gels for Figure 7A

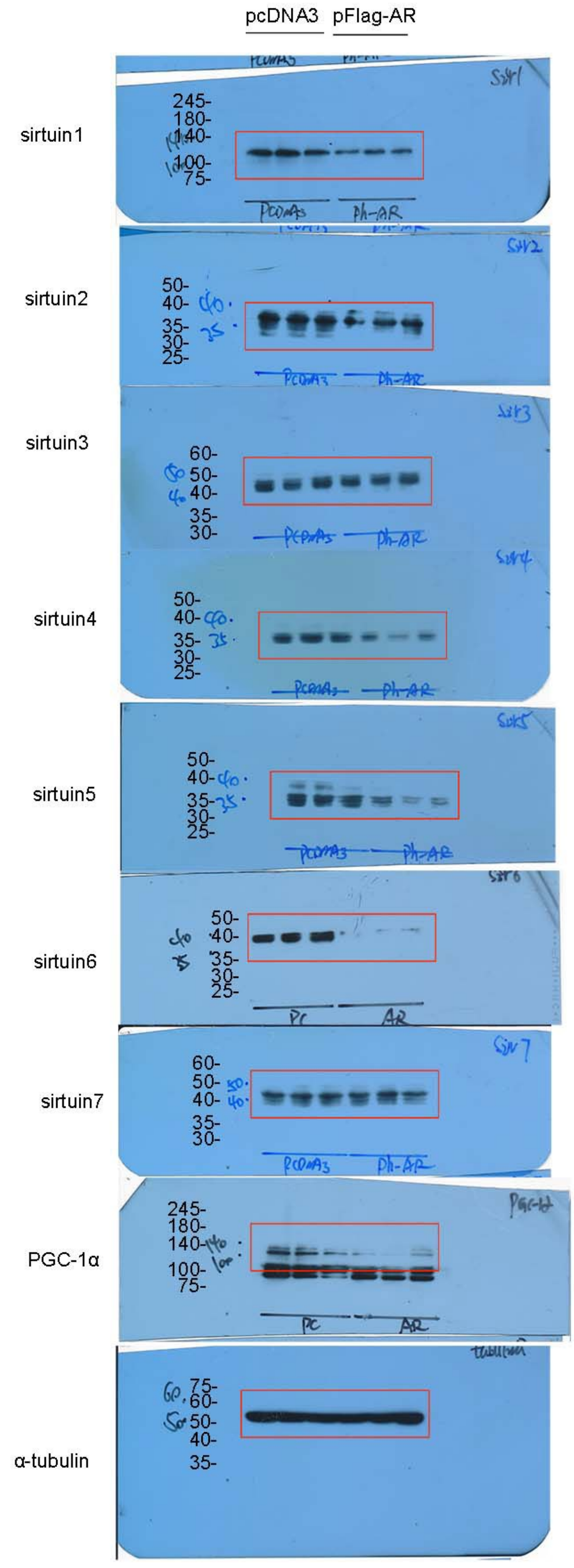

Full unedited gels for Figure 7F

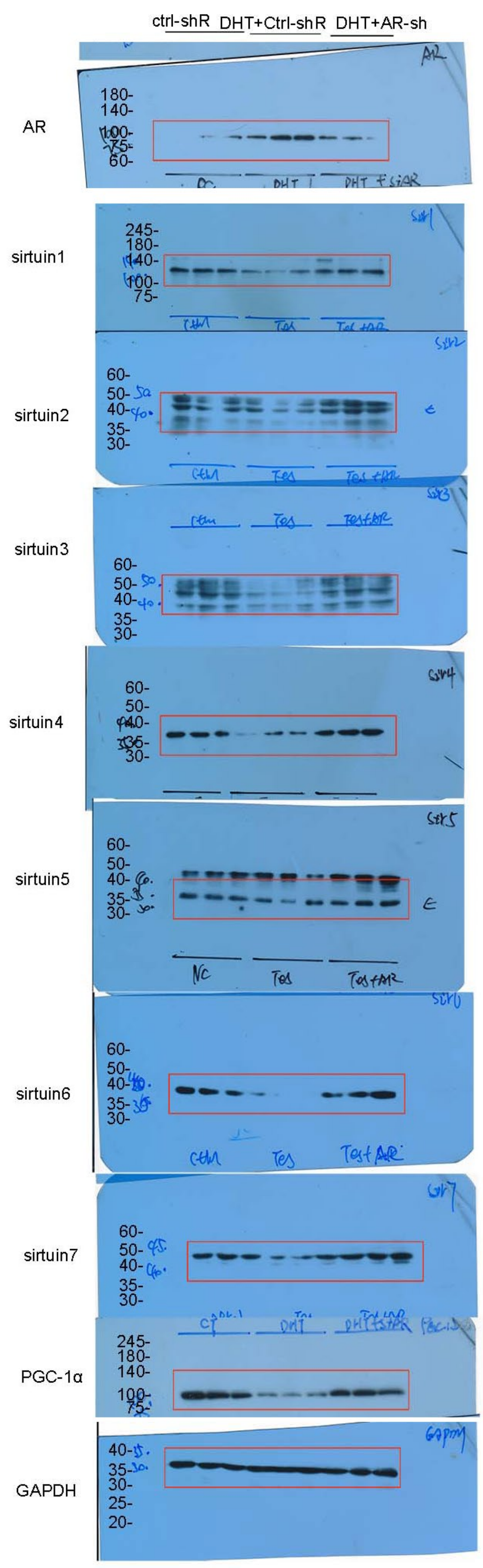

Full unedited gels for Figure 7J

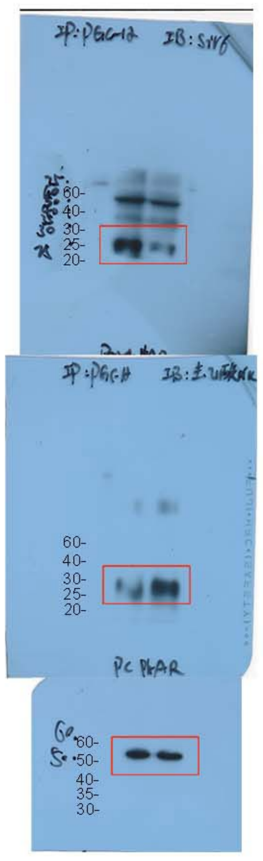

Full unedited gels for Figure 7L

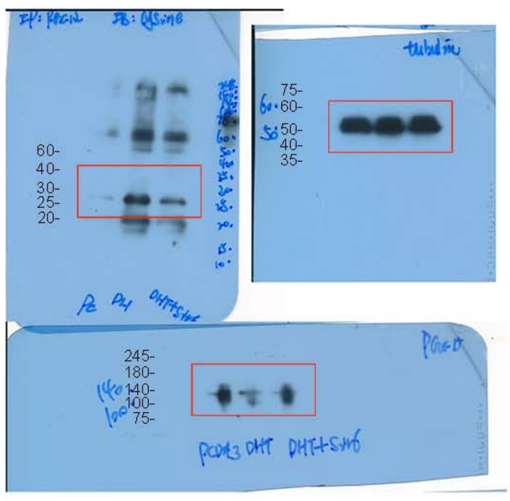

Full unedited gels for Figure 7M

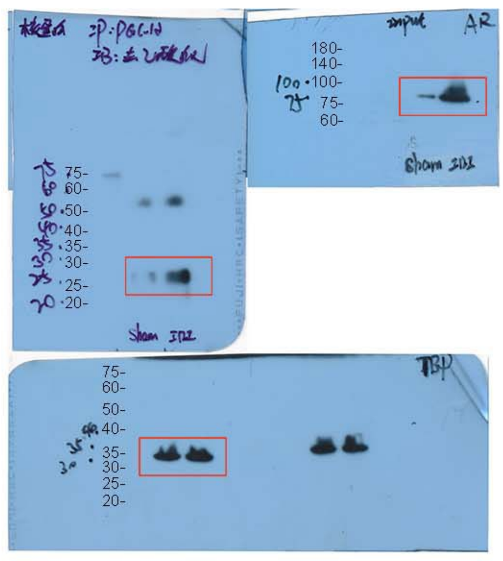

Full unedited gels for Figure 7K

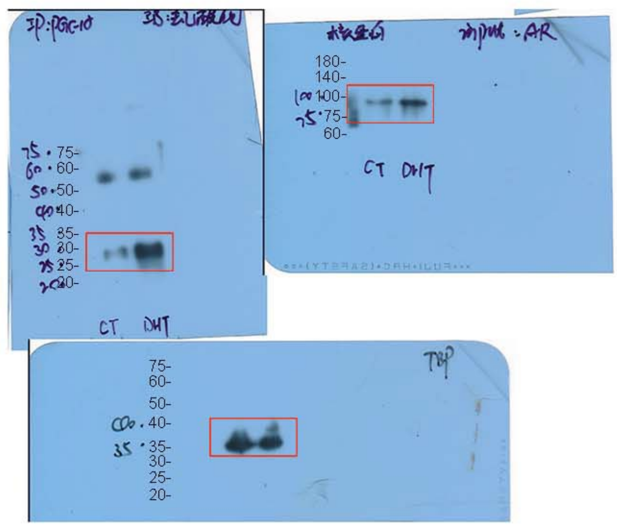

Full unedited gels for Figure 7D

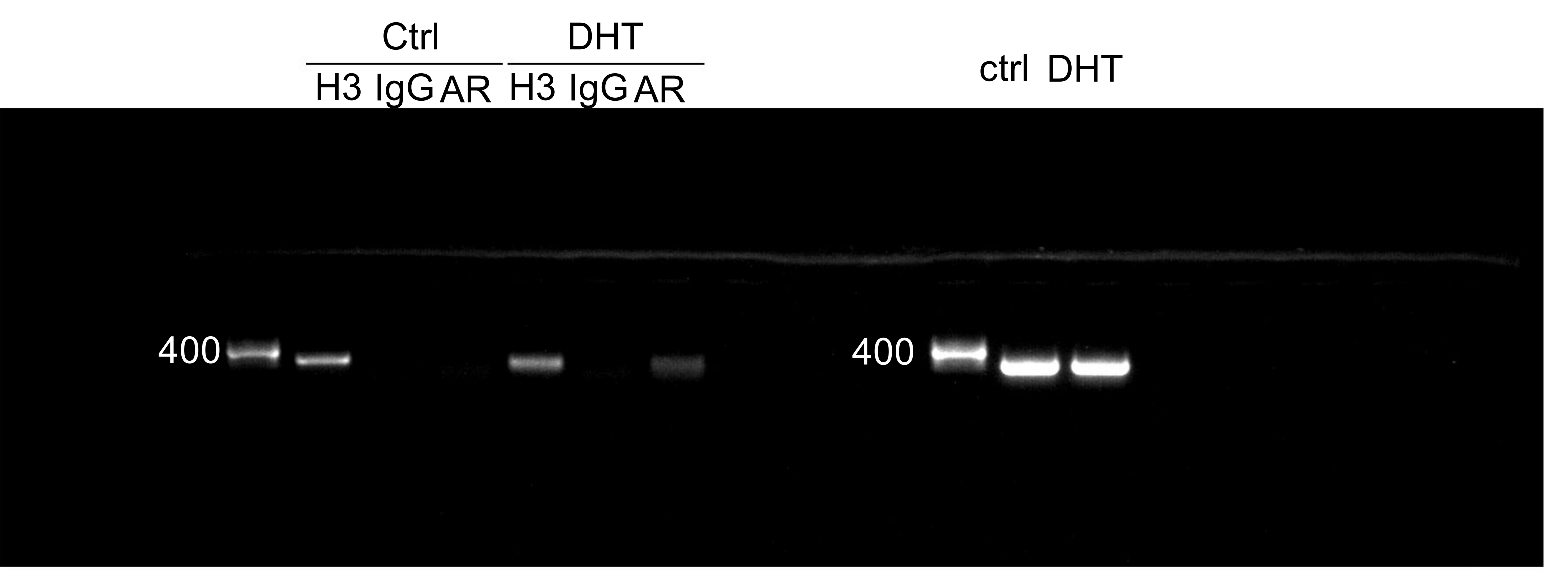

Full unedited gels for figure 8A

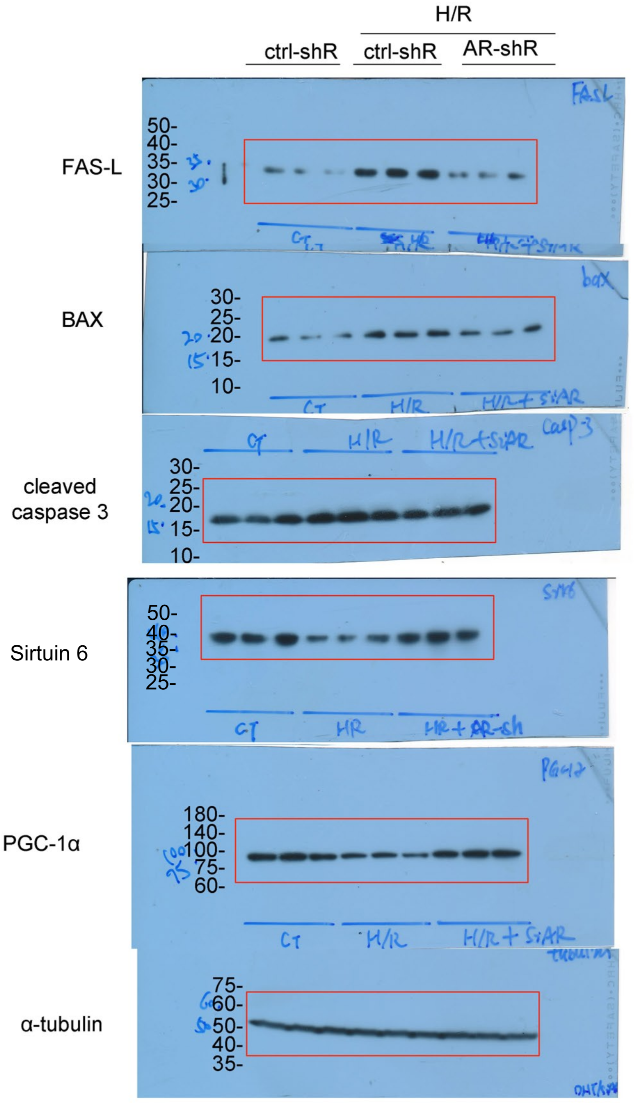

Full unedited gels for figure 8I

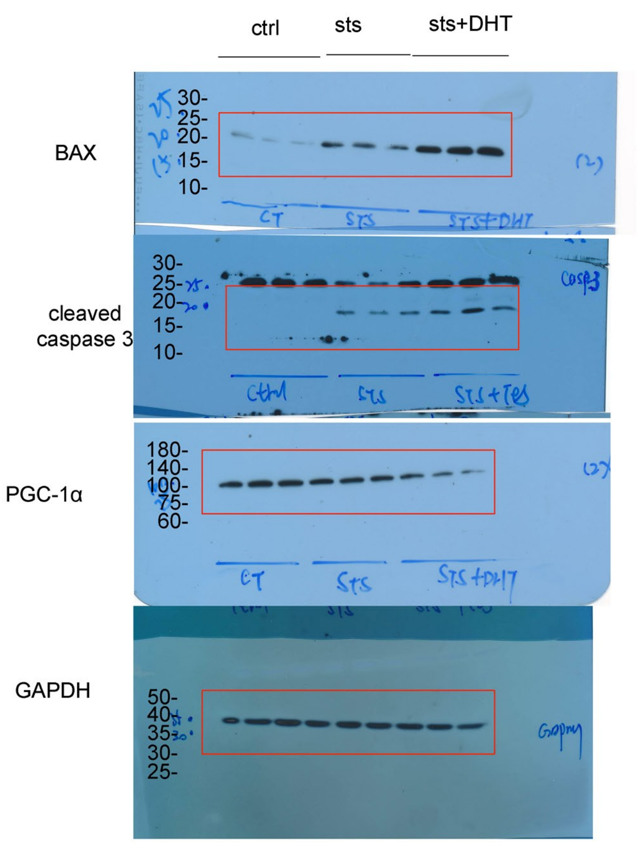

M **Full unedited gels for figure 8M**

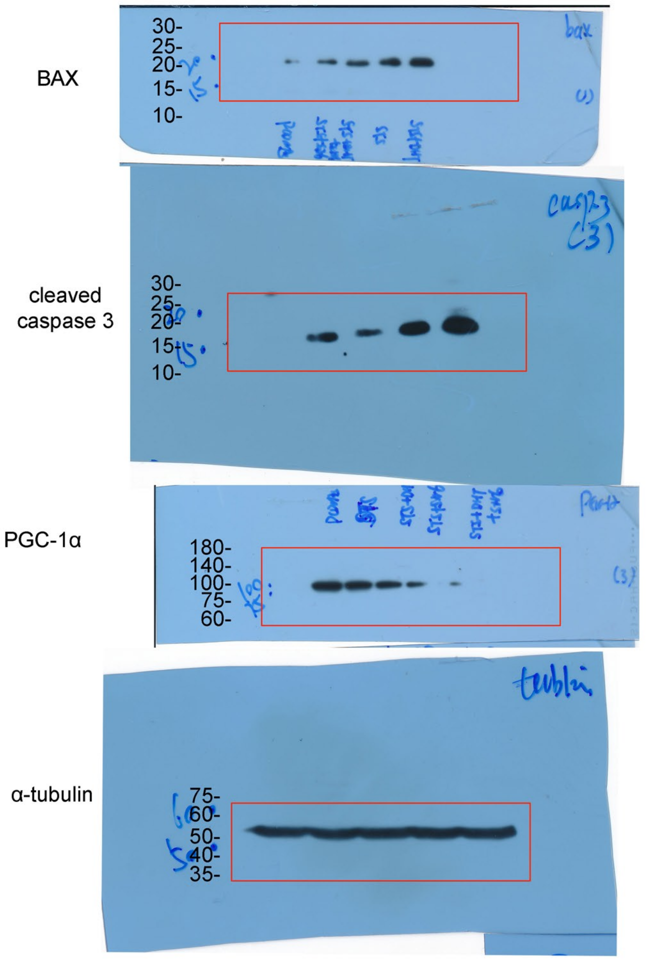

Full unedited gels for supplementary figure1 A

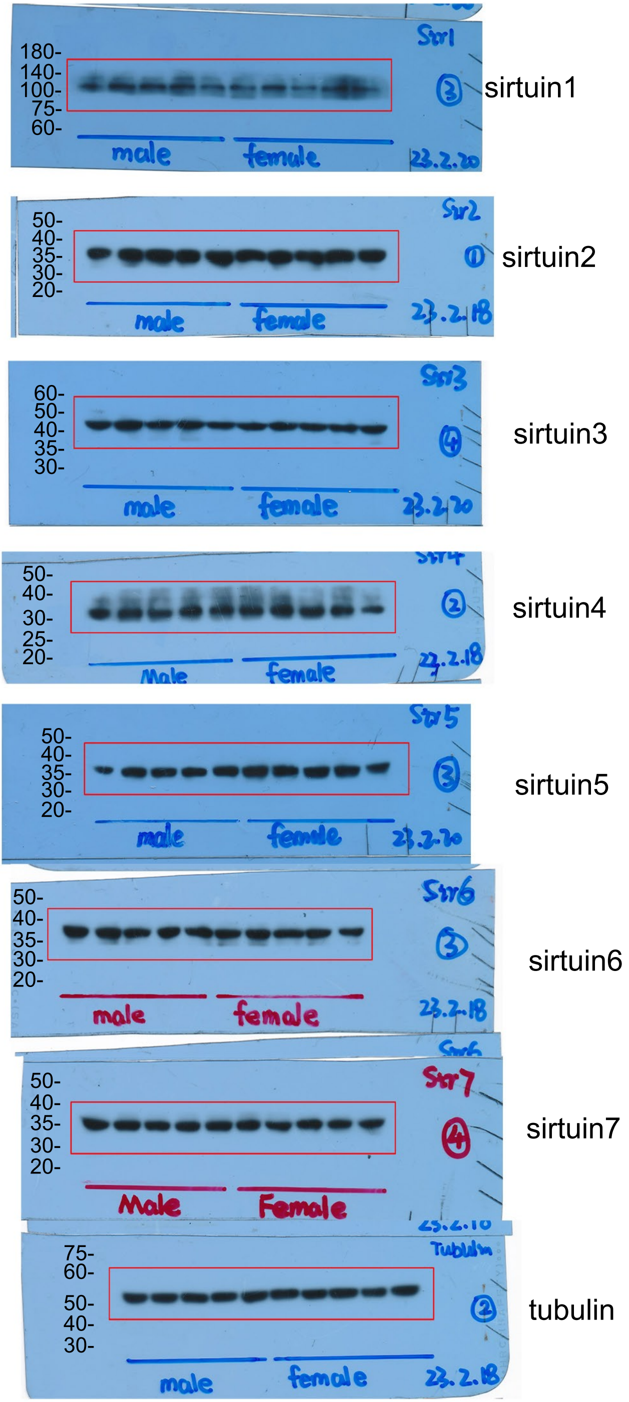

Supplement: Supplementary file 2 — wesrtern blot [file 41420_2023_1432_MOESM2_ESM.pdf]
